# Diagnostic performance of ultra-low-field neurological magnetic resonance imaging: a systematic review and meta-analysis

**DOI:** 10.1136/bmjopen-2026-124306

**Published:** 2026-10-30

**Authors:** Paul Lockwood, Elizabeth Barrett

**Affiliations:** 1 School of Nursing, Midwifery, Allied and Public Health, Canterbury Christ Church University, Canterbury, UK; 2 Radiology, Dartford and Gravesham NHS Trust, Dartford, UK

**Keywords:** Magnetic Resonance Imaging, Neuroradiology, Magnetic resonance imaging, Neuroradiology

## Abstract

**Abstract:**

**Objectives:**

To systematically review the reported level of diagnostic performance of ultra-low-field MRI (ULF-MRI) in brain imaging of both paediatrics (including neonates and infants) and adults from a range of neurological conditions.

**Design:**

A systematic literature review used the Population, Index Test, Reference Test, Diagnsotic Outcome (PIRD) framework to identify keywords for electronic database searches to identify studies of ULF-MRI (index test) against standard cross-sectional imaging (reference standard) that presented diagnostic outcome accuracy, with the search documented in a Preferred Reporting Items for Systematic Reviews and Meta-Analyses (PRISMA) flow chart.

**Data sources:**

A comprehensive literature search was conducted between February and March 2026 in the electronic databases PubMed, Medline, Cumulative Index to Nursing and Allied Health Literature (CINHAL), Embase, Web of Science, Scopus, Cochrane Central Register of Controlled Trials and Google Scholar, without date restriction

**Eligibility criteria:**

Published peer-reviewed primary data studies in English were included, involving human subjects of any age or gender undergoing brain imaging, irrespective of health or clinical indications. Studies were required to have used ULF-MRI, to report diagnostic accuracy or image evaluation and to provide comparative findings with conventional cross-sectional neuroimaging. No restriction on study design, setting, country, patient population or MRI vendor was included. Exclusions included non-brain and non-human imaging, phantom studies, artificial intelligence use or missing diagnostic outcome data.

**Data extraction and synthesis:**

The Quality Assessment of Diagnostic test Accuracy Studies (QUADAS-3) tool was used to assess the quality of retrieved studies. After screening the studies, a meta-analysis for pooled sensitivity, specificity, positive and negative likelihood ratios and diagnostic OR was conducted and expressed in forest plots, pooled heterogeneity values, 95% CIs, probability values and summary receiver operating characteristic curve plotted for area under the curve (AUC).

**Results:**

In total, n=17 studies were identified for pooled meta-analysis; n=9 compared ULF-MRI directly to conventional MRI, and n=8 compared it to a mix of conventional MRI and CT. Wide variability in methodology was identified across pilot and observational studies, with imaging sample sizes ranging from n=8 to n=189, readers n=1–2 and neurological conditions assessed, which affected the risk of bias and warranted caution in interpreting meta-analysis results. Overall sensitivity was 90% (95% CI 0.82 to 0.94); specificity was 98% (95% CI 0.97 to 0.99); AUC was 0.99, with significant heterogeneity (χ^2^=39.77; p=0.008; I2=59.8%) between studies.

**Conclusion:**

Significant heterogeneity in study methodologies was identified, warranting caution when interpreting the meta-analysis results. The studies demonstrated high diagnostic performance for gross structural neurological conditions, but detecting small, subtle lesions below 6 mm was problematic with ULF-MRI. However, ULF-MRI is not positioned in the studies as a replacement for conventional MRI, but rather as a complementary tool for monitoring, triage and imaging follow-up during bedside assessment, particularly in critical patient environments.

**STRENGTHS AND LIMITATIONS OF THIS STUDY**

- This study used the QUADAS-3 framework to assess the methodological quality and risk of bias across the found studies.
- A comprehensive literature search found n=17 studies from PubMed, MEDLINE, CINAHL, Embase, Web of Science, Scopus, Cochrane Central Register of Controlled Trials and Google Scholar.
- Meta-analysis applied pooled sensitivity, specificity, positive and negative likelihood ratios, diagnostic OR and summary receiver operating characteristic curve plotted for area under the curve.
- A key limitation is the substantial heterogeneity in study designs and population samples across included studies, which may have reduced study comparability and limited the generalisability of the pooled findings; therefore, the results should be interpreted with caution.

## Introduction

 MRI is a standard non-invasive diagnostic imaging examination for many anatomical and functional structures of the human body. Image quality is often determined by the magnetic field strength, measured in tesla (T) or millitesla (mT), at the centre of the magnet, and by the frequency of the electromagnetic radio frequency energy pulses used to collect the signal and create an image.^1^

Within this paper, we acknowledge that MRI definitions and terminology vary to some degree within the literature and globally, but the widely accepted classifications are as follows. Ultra-low-field MRI is a scanner with a magnetic field strength of less than 0.1 T (100 mT). An example would include the commercial portable hyperfine scanner^2 3^ with a field strength of 0.064 T, although variations of 0.04 mT have been tested since the 1970s.^4^ However, at this low-field (LF) strength, there are concerns about low signal-to-noise ratio (SNR), background image noise affecting fine anatomical detail (contrast between adjacent tissue and vessel boundaries) and image artefacts that reduce diagnostic quality. LF MRI is a scanner with a magnetic field strength between 0.1 T and 1.0 T. Examples include scanners with a field strength of 0.35T,^5–7^ 0.5 T^8–12^ or 0.7 T.^13 14^ High-field (HF) MRI is a scanner with a magnetic field strength between 1.5T and 3T,^1^ developed in the 1970s,^15–19^ before the first commercial version in the 1980s,^4 20^ and is now used in routine clinical practice. There are also ultra-high-field MRI scanners, with magnetic field strengths of 7 T and above,^1 21^ commonly used in research.

As an imaging modality, ULF-MRI is becoming popular due to its point-of-care design and the low magnetic field strength, which reduces the risk to the surrounding environment. ULF-MRI can be used outside a normal clinical environment (eg, a hospital) to serve hard-to-reach communities for instant imaging^22^ without the high-cost specialist room requirements.^23^ The advantages in point-of-care access to imaging over traditional MRI/radiology department settings include low cost,^24–26^ low-power requirements,^27 28^ portability of equipment between settings^24 25 28^ and ease of staff training.^24 25^ Although concerns remain about risk assessments of patients in the LF magnetic fields,^29^ the majority of existing clinical MRI risk assessment data and evidence^30–32^ (pacemaker safety, metallic implant studies, etc) are written for HF strength MRI scanners (1.5–3T).

For new and emerging imaging technologies to be incorporated into mainstream imaging practices, a robust body of evidence is essential. This evidence should ideally be based on real-world effects of the technology on healthcare decisions and outcomes, demonstrating its value in supporting widespread adoption and scaling. In diagnostic imaging, established hierarchical frameworks^33^ for evaluating the clinical efficacy of imaging modalities have existed since the 1970s,^34^ specifically for MRI is the Mackenzie and Dixon^35^ hierarchical five-stage framework (figure 1) that differentiates each inherent step of MRI performance from technical to diagnostic to the influence on patients’ diagnosis, management and health.

**Figure 1:**
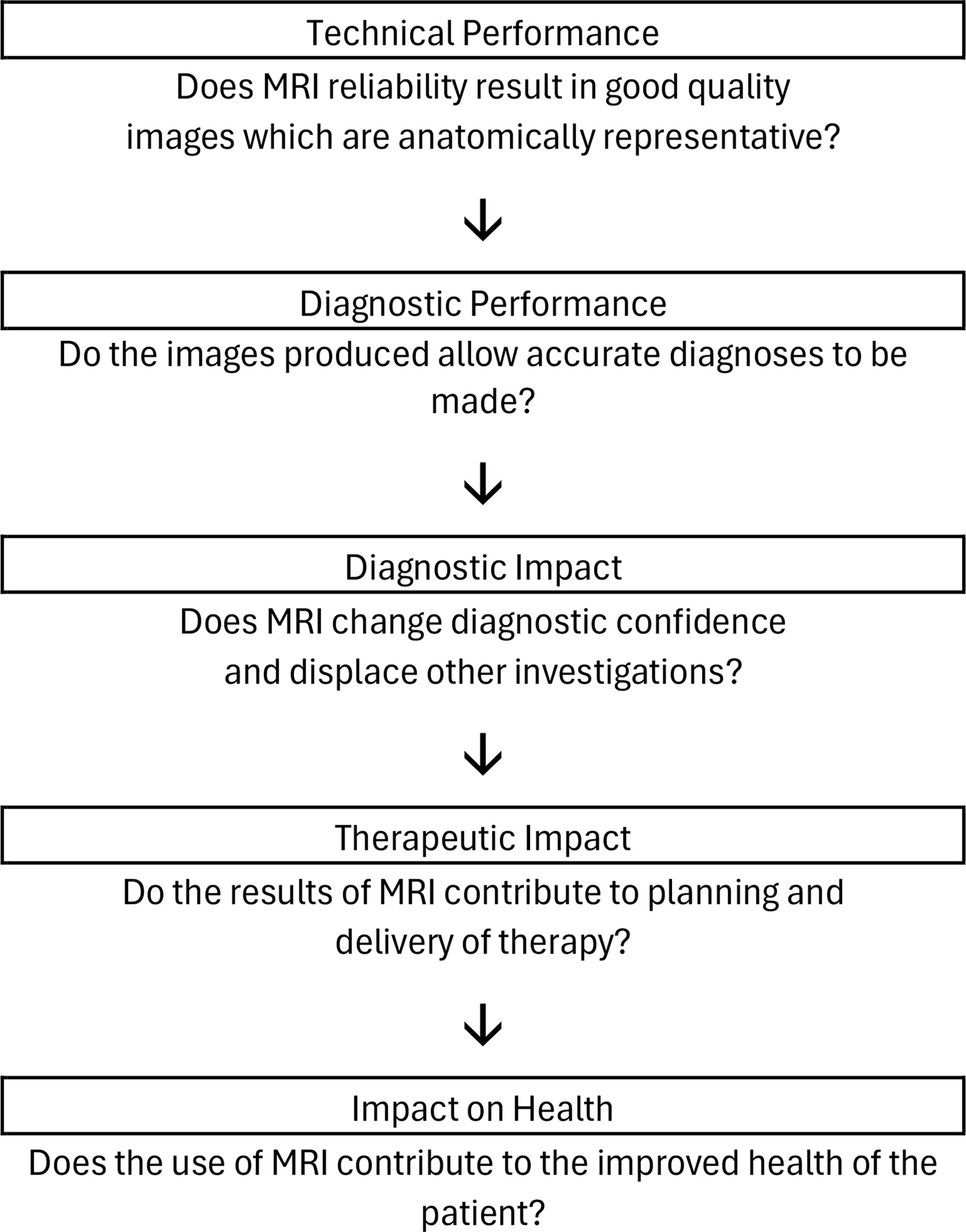
Mackenzie and Dixon^35^ five-stage hierarchical framework to assess the effects of MRI.

Published studies have evaluated the level one^33^ technical efficacy of the early development,^23^ testing and optimisation^36^ of ULF-MRI for neurological imaging. These studies primarily assess imaging datasets to establish outcome metrics for image quality, including SNR, contrast-to-noise ratio and spatial resolution, often incorporating anatomical boundary differentiation,^37^ such as grey-white matter segmentation^38^ or ventricular boundaries^39^ uniformity, image sharpness and artefact severity scoring.^40^ Additionally, research has examined the reproducibility of ULF-MRI in accurately replicating HF-MRI brain volumetric assessments^41^ including anatomical structural measurements for clinical benchmarking and Dice Similarity Coefficients for segmentation overlap and agreement in terms of structural morphometry.^42 43^ With research placing a strong emphasis on volumetric accuracy as an indicator of ULF-MRI’s technical performance and reliability, measuring whole-brain volume, grey/white matter volume, hippocampal volume, cerebrospinal fluid volume or ventricular volume.^22^

This systematic literature review aims to assess the current level two^33 35^ evidence on the diagnostic accuracy of ULF-MRI in detecting neurological conditions and pathologies. The study will not assess the higher Mackenzie and Dixon^35^ or Fryback and Thornbury^33^ clinical efficacy levels of downstream outcomes in the treatment and management of patients nor the benefits to clinical service delivery.

## Methods

### Patient and public involvement

A small peer-review focus group of Patient and Public Involvement and Engagement (PPIE) volunteers was engaged^44–46^ for discussion at the study brainstorming and design stage for review of the topic, theme, outcome measurements to be used and the relevance of the topic to their experience (service users) of improving healthcare access. The PPIE review stage did not generate primary or secondary data for the literature review; it served solely as a voluntary institutional peer-review step of the initial study design. Voluntary participation in the PPIE review focus group was facilitated by a centrally managed PPIE co-ordinator at the study institute, who organised institutional PPIE recruitment, consent and focus group peer-review sessions, with the peer-review focus group held online to reduce PPIE costs and accessibility burdens.

### Identification and selection of studies

The data were obtained from publicly available published studies and did not include individual personal^47^ or patient data,^48^ thereby eliminating privacy concerns in accordance with institutional and national guidelines; formal ethical approval was not required. The review was conducted in accordance with recognised systematic review methodology and relevant reporting standards^49^ (Declaration of Helsinki).^50^

To develop the research question and prepare for the literature search, a PIRD framing^51^ of the question was applied: what is the current evidence on the diagnostic accuracy (D) of neurological (P) ULF-MRI (I) compared with standard cross-sectional imaging modalities (R). The PIRD framing^51^ allowed identification of a combination of keywords (hierarchical controlled vocabularies for health sciences, eg, Medical Subject Headings (MeSH),^52^ free-text terminology and Boolean operators) (table 1; online supplemental material 1).

**Table 1:** PIRD-based search strategy

P population/patient/problem		I index test		R reference test		D diagnostic outcome
('Human patient' OR 'brain' OR 'cerebral' OR 'intracranial' OR 'neurological' OR 'stroke' OR 'tumour' OR 'infection' OR 'haemorrhage' OR 'hemorrhage' OR 'TBI' OR 'Brain Diseases')	AND	('MRI' OR 'magnetic resonance imaging' OR 'ultra low field' OR 'ultra-low-field' OR 'low-field MRI' OR 'portable MRI' OR '0.064T' OR '64mT' OR '0.1T' OR '0.2T' OR 'portable scanner' OR 'Low-cost MRI' OR 'Low-field MRI' OR 'low-field brain MRI' OR 'Point of Care MRI' OR pMRI OR POC MRI OR Mobile MRI OR mMRI OR 'Mobile point of care' MRI OR 'Low Tesla Magnetic resonance imaging' OR 'Low strength Magnetic resonance imaging' OR 'Low strength' MRI OR 'bedside MRI' OR 'bedside portable MRI' OR 'brain only MRI' OR 'head only MRI' OR 'HyperfineSwoop' OR Hyperfine OR 'Halbach MRI' OR Halbach')	AND	('Computed Tomography' OR 'CT' OR 'MRI' OR 'magnetic resonance imaging' OR 'high field' OR 'High-field MRI' OR '1.5T' OR '3T' OR 'reference standard imaging' OR 'standard neurological imaging')	AND	('Diagnosis' OR 'diagnostic accuracy' OR 'sensitivity' OR 'specificity' OR 'agreement' OR 'Positive Predictive Value' OR 'Negative Predictive Value' OR 'Reciever Operating Characteristic' OR 'Area Under the Curve' OR 'AuC' OR 'Probability Values' OR 'p-values' OR 'Likelihood ratios' OR 'Observer performance' OR 'detection') No population terms or condition-specific filters (e.g., treatment outcomes)

The search was conducted between February and March 2026, using the following databases: PubMed, MEDLINE, CINAHL, Embase, Web of Science, Scopus, Cochrane Central Register of Controlled Trials and Google Scholar. All relevant studies published in English were included, with no restriction on publication date.

Eligibility criteria comprised published, peer-reviewed primary data studies without date restriction, involving human subjects of any age or gender undergoing brain imaging, irrespective of health or clinical indications. Studies were required to have used ULF-MRI (index test), to report diagnostic accuracy or image evaluation and to provide comparative findings with conventional cross-sectional neuroimaging modalities such as MRI, CT or other relevant clinical reference standards. Studies were included regardless of country of research, study setting, study design, patient population, MRI manufacturer or vendor or MRI protocol. Exclusion criteria encompassed studies focused on non-brain or non-head imaging (eg, other anatomical regions), phantom studies (physics testing), animal imaging, absence of diagnostic outcome data or the use of artificial intelligence (AI) software for image reading, interpretation or quality enhancement.

### Data extraction process

All retrieved citations were exported to reference management software (Mendeley, V2.131.0; 2025) and an Excel spreadsheet (Microsoft 365; V.2510; 2025). Duplicates were removed, and the final list was prepared for title and abstract screening, full paper review and outcome (accuracy) data retrieval for meta-analysis (Preferred Reporting Items for Systematic Reviews and Meta-Analyses (PRISMA)^49^ flowchart). The process will follow two reviewers independently assessing the literature; disagreements will be resolved through discussion. Risk of bias scores will be used to inform interpretation of results. No studies will be excluded solely based on quality rating.

### Assessment of methodological quality

The National Health Service (NHS) applies evidence-based research to determine patient risk, thereby informing effective treatment pathways and the cost-effectiveness of interventions.^53^ In the appraisal of any healthcare intervention, the most effective approach is a balanced framework that reduces bias, acknowledges uncertainty in the data and applies rigour and validity to the process. The Medical Research Council^54^ promotes a systematic approach to planning multifactorial, evidence-based investigations and assessments in healthcare development. Each study identified for inclusion was independently assessed for the risk of bias with the QUADAS-3^55^ tool with disagreements resolved through discussion.

### Data analysis

A thematic matrix of found studies will present: author’s name, year of study publication, country, study design, imaging intervention applied (ULF-MRI equipment and protocol), comparator reference standard imaging applied (if given), setting (community, laboratory, hospital, etc), sample size, diagnostic accuracy metrics, level of evidence and adverse events reported.

For statistical analysis, missing data in reporting studies were sought from the study authors. If sufficient data could not be obtained, we excluded those studies from the data synthesis. Where feasible, diagnostic accuracy using 2×2 contingency tables (true positive (TP), true negative (TN), false positive (FP), false negative) was evaluated from reported sample sizes and outcome measures to confirm results, including all participants in the groups to which they were originally assigned for sensitivity (TP rate), specificity (TN rate), accuracy (detection of pathology) and agreement (comparison with a reference standard for the same imaging modality). Meta-analyses used Meta-DiSc^56^ software (V.1.4; 2006) to produce separate univariate meta-analyses forest plots of pooled overall sensitivity and specificity (DerSimonian-Laird random-effects models).^56^ For clinical importance, additional metrics comprised positive likelihood ratios (PLR) and negative likelihood ratios (NLR) for the probability of having or not having a condition given a test result, were conducted to summarise the reported ULF-MRI probability for diagnosis and the impact of heterogeneity between studies that affects this outcome. Pooled inconsistency between study results (I²) due to variation, with *χ*^2^ for heterogeneity (consistency of studies), with probability values (p values; <0.05) and 95% CI (95% CI) range of values for true effect size. With a summary diagnostic odds ratio (sDOR) for overall how well ULF-MRI in the studies discriminated between patients with and without neurological conditions. With a receiver operating characteristic (SROC) curve plotting with area under the curve (AUC) using Moses–Littenberg method^56^ as a global metric of the multiple studies’ diagnostic ability. To account for differences between study results, a diagnostic threshold analysis was conducted using the Spearman correlation coefficient (r_s_) to assess the strength and direction of association across values, sensitivity and specificity thresholds and Tau squared (τ²) to quantify unexplained between-study variance.

## Results

### Study selection

A total of n=17 studies were identified for inclusion in the review (figure 2). All studies^36 57–72^ provided a range of evidence from prospective diagnostic accuracy/technical feasibility observational studies to retrospective cohort studies, applying the same ULF-MRI model.^2^

**Figure 2:**
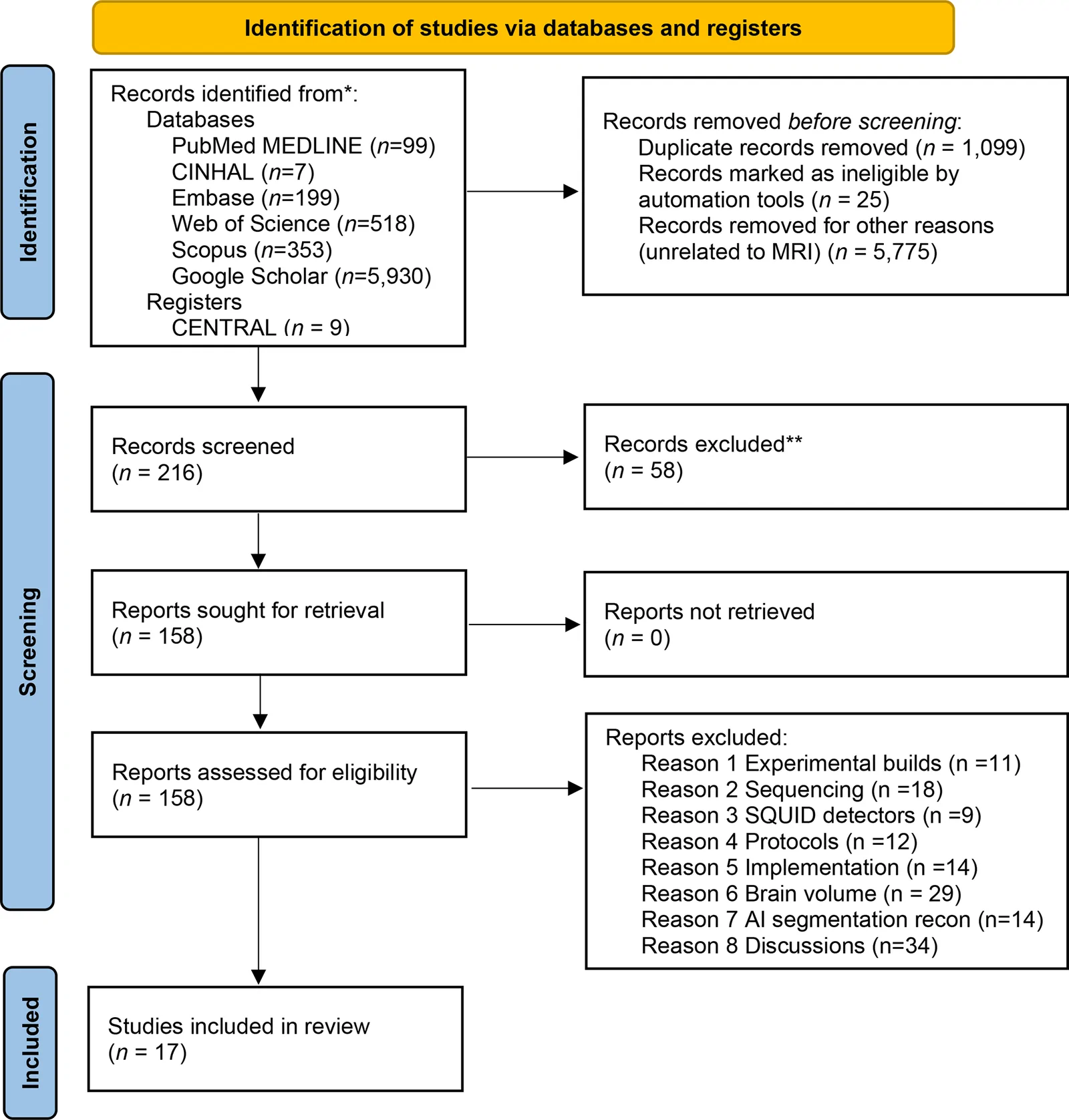
Preferred Reporting Items for Systematic Reviews and Meta-Analyses (PRISMA)^49^ flowchart of identified and filtered studies. AI, artificial intelligence; CENTRAL, Superconducting Quantum Interference Device; SQUID, Cochrane Central Register of Controlled Trials

### Characteristics of included studies

However, there were variations to the study design, including n=9 studies^36 57–61 64 67 68^ comparing directly to conventional MRI scanners (1.5T/3T/7T) and n=8 studies comparing to a mix of MRI (1.5T/3T) and CT.^62 63 65 66 69–72^ In addition, only n=9 of the studies^36 57–59 61 64 67 68 70^ reported the MRI imaging protocols and sequences applied.

The selection of studies included a wide sample size of patients’ scans ranging from n=8^60^ to n=189,^66^ with a mix of neurological pathological conditions detected. Only one study by Cho *et al*^72^ reported adverse events in n=3 patients during ULF-MRI imaging related to interference of the magnetic field with surrounding medical equipment, all of which were temporary and resolved at the patient’s bedside, with no lasting harm or risk reported.

### Quality appraisal

Risk of bias was assessed^55^ for all included studies (online supplemental material 2), which demonstrated variability from low,^62 67^ low to moderate,^57–59 61 64 65 70 72^ moderate,^69 71^ moderate to high^36 66^ and high^63 68^ risk of bias due to the methodology applied. Overall risk of bias was influenced by inconsistent reference standards of comparator imaging applied (1.5T/3T/7T MRI and CT) and missing technical parameters, sequences for comparator imaging, flow and timing of imaging and patient selection (sample size, limitations of neurological conditions reviewed) and unblinded reporting of data influenced heterogeneity across studies.

### Meta-analysis of included studies

Statistical analysis of the studies (n=17) using forest plots for sensitivity (figure 3) provided a visual display of the reported findings and sample sizes across all studies, with a pooled sensitivity of 90% (95% CI 0.82 to 0.94), evidencing the reported ability of ULF-MRI to detect pathological change on imaging. However, there was significant heterogeneity (χ^2^=39.77; p=0.008; I^2^=59.8%) between studies (eg, sample sizes and disease conditions). This inconsistency and variation across clinical studies warrant caution in interpreting findings at this early stage of ULF-MRI research.

**Figure 3:**
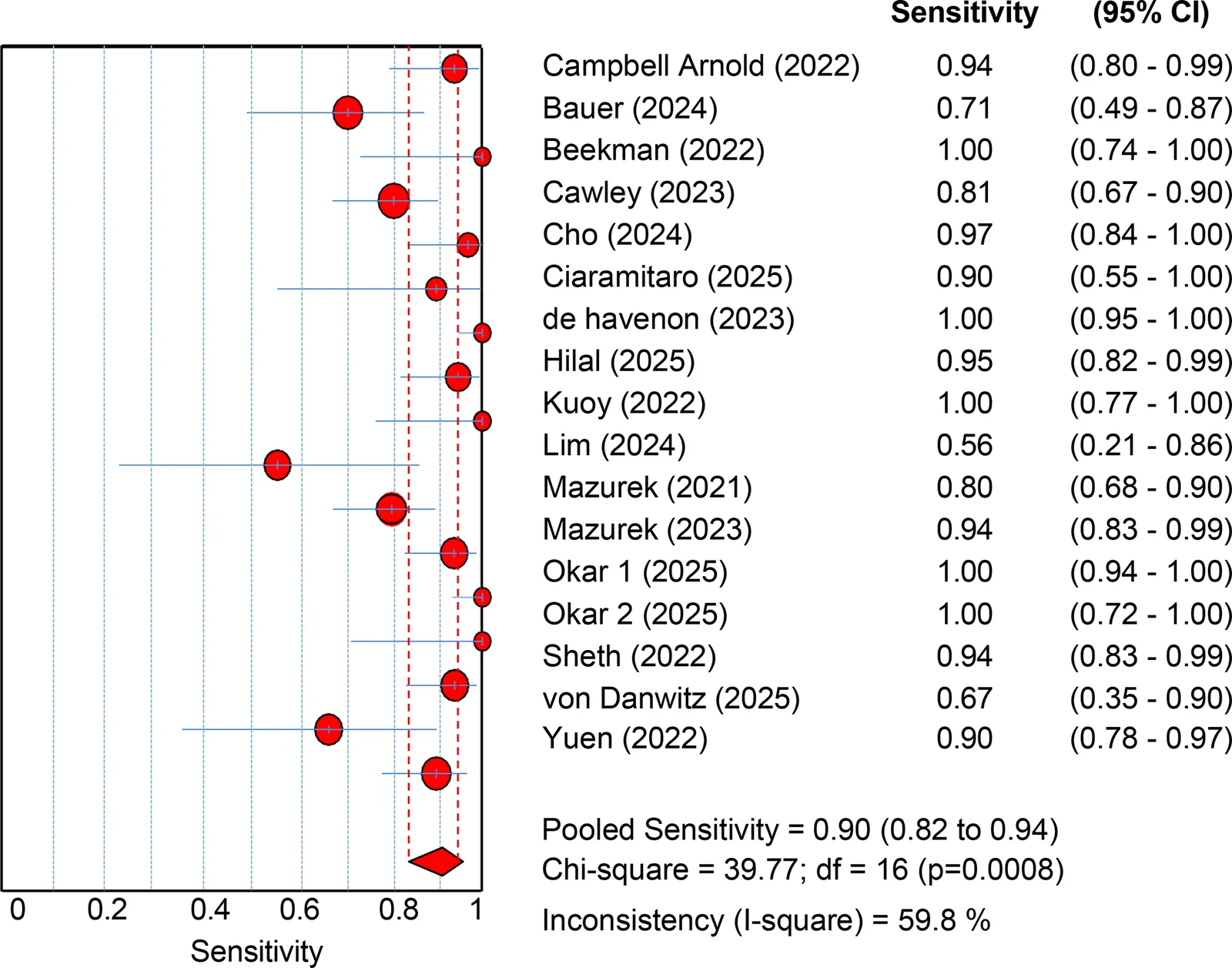
Forest plot of the sensitivity of neurological ultra-low-field magnetic resonance imaging (ULF-MRI) in the included studies.

It was noted that some diagnostic studies^57 58 60 67 68 71^ had no normal patients (zero cell counts in the columns TN/FP), which affected specificity calculations and the inclusion of studies (n=11/17) in the meta-analysis for specificity (figure 4). However, the pooled specificity of ULF-MRI within the included studies (n=11/17) evidenced a high specificity (98%; 95% CI 0.97 to 0.99), which was consistent across the included studies (χ^2^=17.38; p=0.0663; I^2^=42.5%).

**Figure 4:**
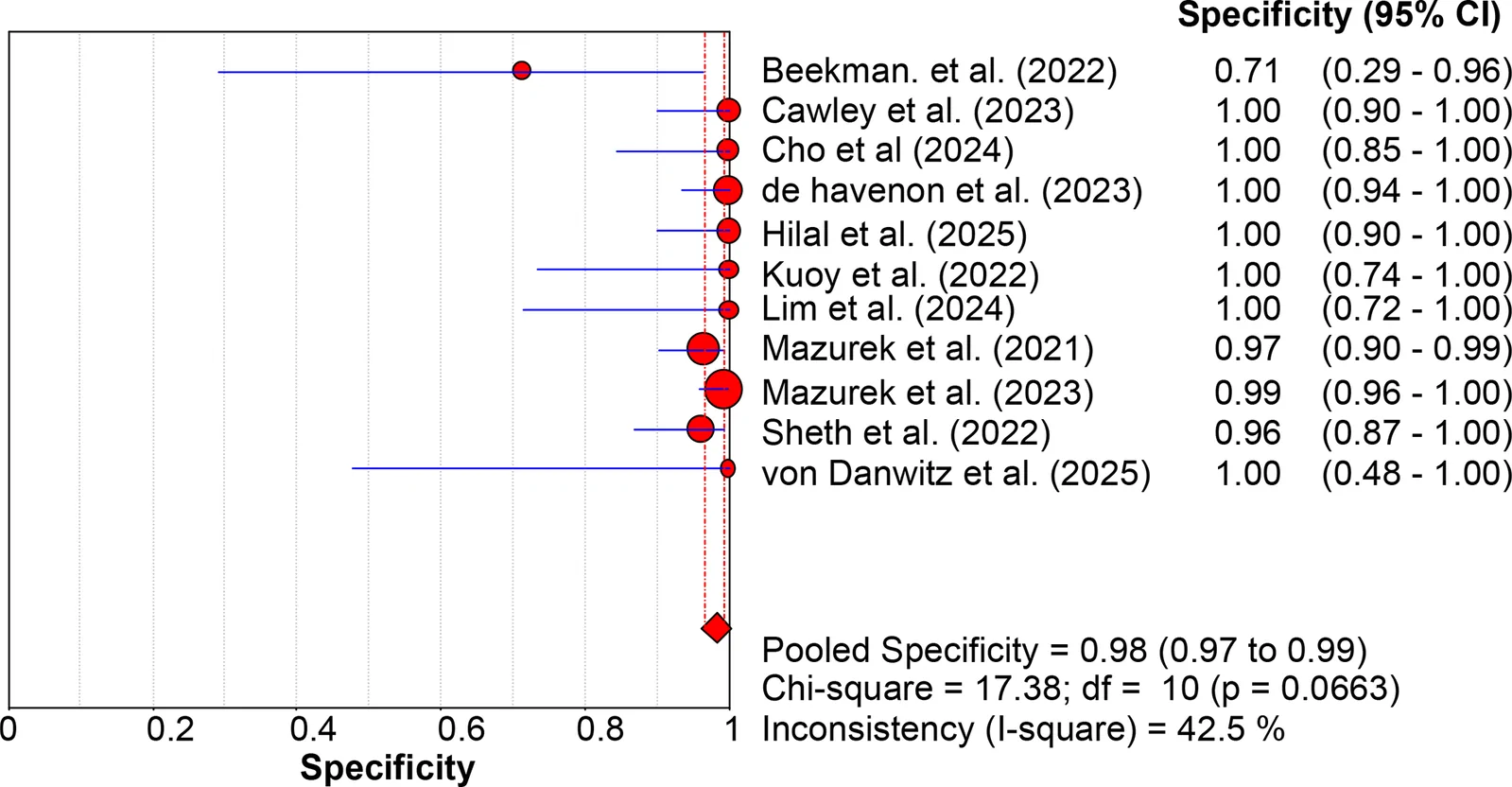
Forest plot of the specificity of neurological ultra-low-field magnetic resonance imaging (ULF-MRI) in the included studies.

Combining the sensitivity and specificity data from n=10/17 studies^36 59 61–66 69 72^ (due to data availability reported in studies) into a SROC plot of reported ULF-MRI diagnostic accuracy as a global performance summary indicates a strong probability of its performance (AUC=0.99; figure 5).

**Figure 5:**
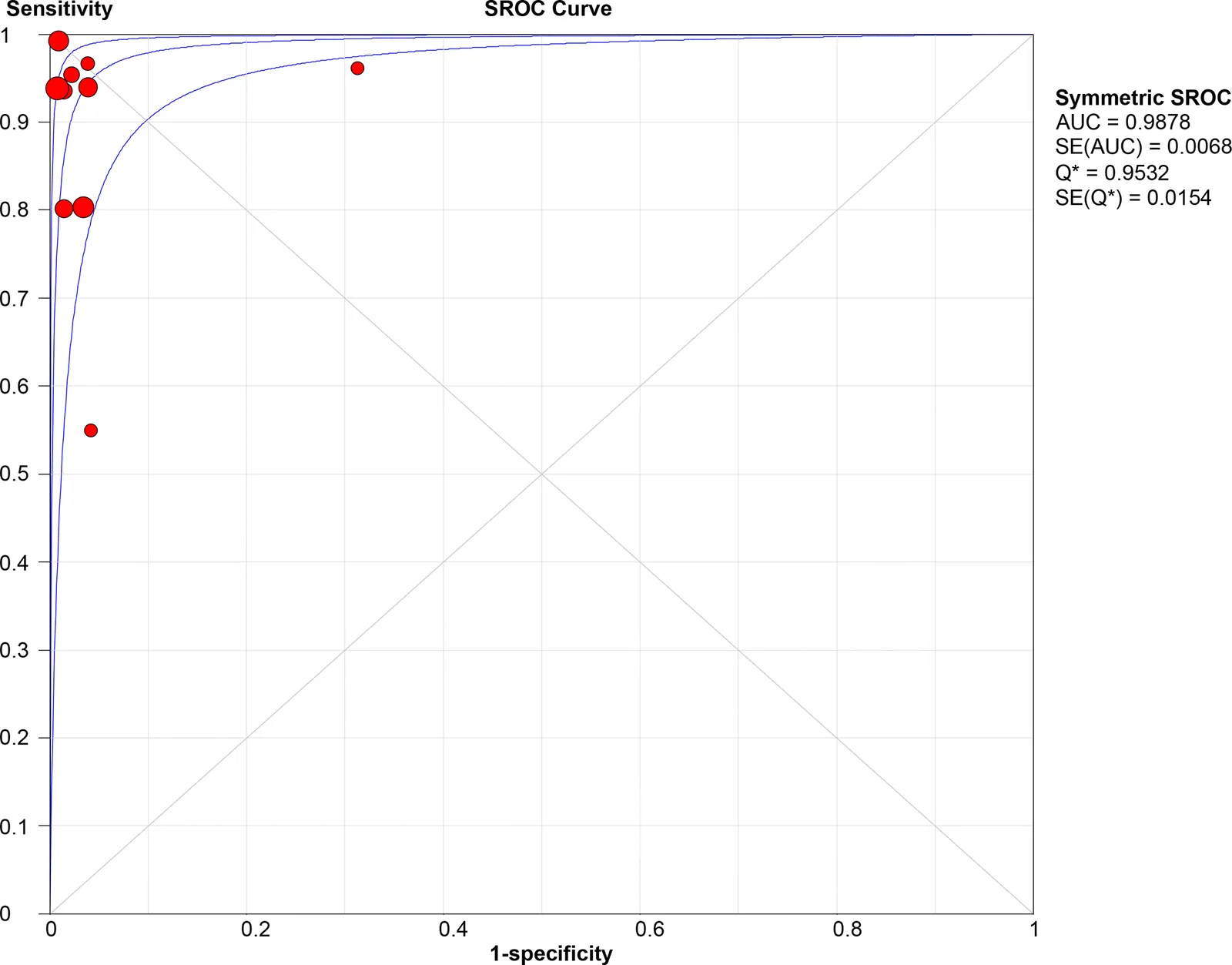
Summary receiver operating curve (SROC) with area under the curve (AUC) calculated for neurological ultra-low-field magnetic resonance imaging (ULF-MRI) in the included studies.

The PLR and NLR assessed the clinical effectiveness, with a summary PLR of 27.48 (95% CI 10.15 to 74.34) pooled across n=10/17 studies^36 59 61–66 69 72^ (due to data availability reported in studies) presents an overall strong diagnostic performance of ULF-MRI for the effectiveness of confirming a positive test result when neurological conditions are present. However, there was some uncertainty in the findings (χ²=23.88; p=0.004; I²=62.3%) due to moderate to substantial variance (heterogeneity) between studies (sample sizes, study designs and methods, conditions, etc); thus, the random-effects model was applied for analysis.

The summary NLR of 0.100 (95% CI 0.050 to 0.201) was pooled across n=10/17 studies^36 59 61–66 69 72^ (due to data availability reported in studies) and demonstrated a strong ability to exclude neurological conditions when normal ULF-MRI scans are reviewed, but with the high variability between studies (χ²=38.38; p=0.00; I²=76.5%), the lack of consistency across studies (sample sizes, study designs and methods, conditions, etc) again recommends caution with these findings.

Combining both sets of data (sensitivity and specificity), an sDOR using a random-effects model was pooled across n=10/17 studies^36 59 61–66 69 72^ (due to the quality of reported data), indicated an overall consistently high diagnostic performance (sDOR=369.23; 95% CI 134.72 to 1011.9) of ULF-MRI, accounting for variance between studies (*x*^2^=13.35; p=0.147; I²=32.6%). It was noted that the differences between studies were not due to diagnostic thresholds applied within individual studies (r_s_=−0.024; p=0.947; τ2=0.9319) but rather to study design, sample size, etc.

### Key themes

The studies demonstrated high diagnostic accuracy for gross structural neurological conditions and strong specificity. But sensitivity for small and subtle lesions below 6 mm was problematic to detect,^36 57 58 60 64 68 70 71^ potentially due to the reduced spatial resolution and SNR inherent in ULF-MRI imaging. The studies consistently underscored the feasibility and advantages of using ULF-MRI outside traditional radiology departments and across different clinical environments, such as intensive care units (ICUs) and stroke wards. This benefits patient access by reducing critical patient transport and clinical risk to health. However, there was little explanation of where the image reporting occurred, whether it is in radiology departments or elsewhere, will impact the advantage of ULF-MRI for diagnosis. Most of the studies identified were pilot or feasibility studies, which impacted the level of evidence (table 2) and risk of bias (online supplemental material 2), or had small sample sizes^58–60 64 68 70^ with methodological issues in recruitment of patients (convenience sampling of ICU or stroke inpatients) or limited numbers of pathological cases.^36 57 59–61 64 67 68 70 71^

**Table 2:** Summary of included studies

Author (year)	Setting and design	ULF-MRI	Comparator	Sample and pathology	Readers	Findings	Level of evidence and adverse events
Arnold *et al*^57^ (2022)	USA; prospective cross-sectional	Hyperfine 64 mT (T1w FSE, T2 FLAIR, T2w FSE)	3T Siemens MRI (T1w MPRAGE, T1w FSE, T2w, T2 FLAIR FSE, T2w FSE)	n=33 adults; MS	n=2 NR	94% sens for WMH (min. 6 mm), false-positive in venous sinus and subcortical artefacts.	Level III; none
Bauer *et al*^58^ (2024)	Germany; prospective cross-sectional	Hyperfine 64 mT (T1w, T2w, FLAIR, DWI)	3T Philips MRI (FLAIR, T1w, T2w, DWI)	n=23 adults; epilepsy, Lsn, MCD, gliosis, atrophy, PTI	n=2 NR	n=24 pathologies in n=23 patients, subtle pathologies missed cysts, microbleeds. 71% sens	Level III; none
Beekman *et al*^59^ (2022)	USA; retrospective observational diagnostic accuracy (single-centre, consecutive cohort)	Hyperfine 64 mT (FLAIR, T1, T2, DWI/ADC)	Conventional MRI (FLAIR, T1, T2, DWI/ADC)	n=19 adults; cardiac HIBI	n=1 NR	n=22 scans from n=19 patients, 100% sens for HIBI	Level III; none
Cawley *et al*^36^ (2023)	UK, USA; prospective, single-centre cohort study	Hyperfine 64 mT (T1w FSE, T2w FSE)	3T Philips MRI (T1w MPRAGE, T2w)	n=87 infants; INF, HIBI, CM	n=1 NNR	n=87 infants (n=102 scans) missed WMH (punctate Lsn), cystic LSn, thalamus ICH	Level III; none
Cho *et al*^72^ (2024)	USA; prospective observational multicentre	Hyperfine 64 mT (FLAIR, T1w, T2w, DWI, ADC)	HF-MRI (n=18); CT (n=36) (sequences not reported)	n=50; ABI, IS, ICH, HIBI	n=2 NR	ULF-MRI observed n=8/10 ABI, n=1/2 for ICH	Level III; n=2 minor, n=1 serious
Ciaramitaro *et al*^60^ (2025)	USA; prospective observational diagnostic study (single centre)	Hyperfine 64 mT (FLAIR, T1w, T2w, DWI)	HF-MRI (Sequences not reported)	n=8; IS	N/A	Smallest INF 4.5 mm, missed a 3.6 mm INF, 67.5% sens, 96.2% spec, PPV 96.4%, NPV 65.8%	Level IV; none
de Havenon *et al*^61^ (2023)	USA; retrospective and prospective observational cohort (diagnostic performance)	Hyperfine 64 mT (sequences not reported)	1.5T MRI (FLAIR)	n=124 adults; WMH	n=3 NR	Retrospective n=16/33 WMH. Prospective n=53/91 WMH. Inter-rater agree k=0.81, inter-modality (MRI) k=0.66	Level III; none
Hilal *et al*^62^ (2025)	Pakistan; prospective, single-centre diagnostic accuracy	Hyperfine 64 mT (FLAIR, T1w, T2w, DWI, ADC)	HF-MRI; CT (sequences not reported)	n=73; HC, INF, ICH, oedema, MLS, Lsn, SDH, EDH	n=2 PR	HC detection 73% sens, 98% spec, 94% agree with MRI/CT (k=0.8) interobserver agree 90% (k=0.81)	Level II; none
Kuoy *et al*^63^ (2022)	USA; retrospective, single-centre diagnostic accuracy	Hyperfine 64 mT (FLAIR, T1w, T2w)	HF-MRI; CT (sequences not reported)	n=36; IS, ICH, Lsn, HC	n=1 NR	Acc (n=14/36), non-diagnostic examinations (n=10/36)	Level IV; none
Lim *et al*^64^ (2024)	Canada; retrospective, single-centre diagnostic accuracy	Hyperfine 64 mT (T2 FLAIR, T2 FSE)	3T Siemens MRI (T2 FLAIR, T2 FSE)	n=20; ON, MS	n=2 NR	Interrater agree 80% (k=0.58) (Lsn 75–90%; k=0.42-80), sens 56%, inability to detect small Lsn	Level III; none
Mazurek *et al*^65^ (2021)	USA; prospective, single-centre diagnostic accuracy	Hyperfine 64 mT (T2w FSE, T2w FLAIR FSE)	1.5/3T Siemens MRI; CT (sequences not reported)	n=144; ICH, IS	n=2 NR	ICH volumes on ULF-MRI, sens 80.4%, spec 96.6%	Level III; none
Mazurek *et al*^66^ (2023)	USA; prospective, single-centre diagnostic accuracy	Hyperfine 64 mT (T1w, T2w, FLAIR, DWI, ADC)	1.5/3T Siemens MRI; CT (sequences not reported)	n=189; ICH, IS, SAH	n=3 NR	AI recon improved acc 97.9%, sens (92.1%), spec (99.3%) for ICH	Level III; none
Okar *et al*^67^ (2025)	USA; retrospective and prospective, single-centre diagnostic accuracy	Hyperfine 64 mT (T1w FSE, T2 FLAIR)	3T Siemens MRI (T1w FSE, T1w MP2RAGE, T2w FLAIR, T2w EPI, T2w FSE, PD-T2w)	n=55; MS	n=2 NR	Periventricular WMH identified, low spec, no normal cases in study	Level III; none
Okar *et al*^68^ (2025)	USA; prospective, single-centre diagnostic accuracy	Hyperfine 64 mT (T2 FLAIR, T1w, DWI, T2w)	3T Philips/3T Siemens/7T Siemens MRI (T2 FLAIR, T1w MPRAGE, DWI, PD-T2w)	n=11; PML	n=2 NR	Sens 62% varied by Lsn conspicuity (punctate or subtle), no normal cases in study	Level IV; none
Sheth *et al*^69^ (2022)	USA; prospective, single-centre diagnostic accuracy	Hyperfine 64 mT (T2w, T1w, FLAIR, DWI)	HF-MRI; CT (sequences not reported)	n=102; IS; ICH	n=3 R	Agree k=0.87 for MLS, sens 93%, spec 96%	Level II; none
von Danwitz *et al*^70^ (2025)^70^	Germany; prospective, single-centre diagnostic accuracy	Hyperfine 64 mT (FLAIR, DWI)	1.5T (n=4)/3T (n=11) Philips MRI (T1w, MRA, FLAIR, DWI)/CT (n=2)	n=17; IS	n=1 NR; n=1 NVA	Low sample, INF smaller than 6 mm not detected, 67% sens	Level II; none
Yuen *et al*^71^ (2022)	USA; prospective, single-centre cohort study	Hyperfine 64 mT (T2w, FLAIR, DWI, ADC)	1.5T/3T MRI; CT (sequences not reported)	n=50; IS	n=2 NS	INF as small as 4 mm detected, 90% detection rate reported (n=45/50)	Level II; none

Pathology: MS, MCD, PTI, HIBI, CM, ABI, IS, ICH, WMH, SDH, EDH, HC, INF, MLS, Lsn, SAH, ON, PML. Readers: NR, NNR, PR, R, NVA, NS. Findings: sens, spec, acc, agree.

ABI, acute brain injury; acc, accuracy; agree, agreement; CM, congenital malformations; EDH, epidural hematoma; HIBI, hypoxic ischaemic brain injury; ICH, intracerebral haemorrhage; IS, ischaemic stroke; MCD, malformations of cortical development; MS, multiple sclerosis; NNR, neonatal neuroradiologist; NR, neuroradiologist; NS, neuro scientists; NVA, neurovascular attending; PR, paediatric radiologists; PTI, post traumatic Injury; R, researchers; SDH, subdural hematoma; sens, sensitivity; spec, specificity; WMH, white matter hyperintensity.

## Discussion

This systematic literature review’s findings of the level two^33 35^ evidence on the diagnostic performance of ULF-MRI demonstrate a baseline performance through meta-analysis and synthesis of published metrics of detecting neurological conditions and pathologies. However, ULF-MRI is not positioned within the studies as a replacement for conventional HF-MRI (1.5T/3T), but rather as a complementary tool for monitoring, triage and imaging follow-up during bedside assessment, particularly for critical patients (neonatal units, adults in ICU or stroke wards). The clinical implications are that it may have a downstream patient impact primarily on clinical decision-making within a limited set of studied neurological conditions by identifying clinically actionable conditions or changes to known conditions, rather than primary first-line diagnosis.

Heterogeneity was observed in the threshold effect differences from the sensitivity and specificity pooled estimate scores of studies. The risk of bias assessment^55^ revealed methodological differences across studies that may have contributed to heterogeneity in the diagnostic accuracy estimates, particularly through variations in patient selection, reference standard and flow and timing domains. Sample sizes of patients varied considerably from small pilot or feasibility studies of single centres with convenience or consecutive cohorts, or only patients with known pathology were included, with no normal controls (spectrum/selection bias) inflating sensitivity and limiting specificity scores. Studies assessed a wide range of neurological conditions; these conditions differ markedly in lesion size, lesion conspicuity and imaging requirements, which may influence the diagnostic performance of ULF-MRI. Lower sensitivities were consistently reported in studies where lesions were small, subtle, punctate or cortical in location. Multiple studies specifically noted reduced detection of lesions measuring less than 4–6 mm. These findings suggest that lesion size and conspicuity had an important influence on pooled estimates of diagnostic performance.

The variation in comparator reference standards *n*=9^36 57–61 64 67 68^ with conventional HF-MRI had inconsistent imaging acquisition protocols reported, whereas several others used a combination of MRI and CT^62 63 65 66 69–72^ as reference standards. HF-MRI systems have greater sensitivity for detection of subtle neurological abnormalities, including early ischaemic changes, small infarcts, demyelinating lesions and other parenchymal pathologies, whereas studies using CT as the reference standard may underestimate lesion detection due to lower sensitivity than HF-MRI (potential misclassification, verification or confirmation bias) for certain pathologies such as small ischaemic lesions, white matter disease and demyelinating pathology. This variation in reference standards may have influenced estimates of diagnostic accuracy and contributed to between-study variability. The index test of ULF-MRI demonstrated a lower risk of bias as most studies used predefined protocols (table 2). However, variation was identified in detailed reporting, reader blinding or image interpretation procedures. For flow and timing, some studies did not clearly report intervals between ULF-MRI and comparator imaging. This is particularly relevant in acute stroke and critical care populations where pathology can evolve rapidly.

To assess the potential upscale of ULF-MRI into the routine clinical pathway, the baseline performance of ULF-MRI diagnostic accuracy would need to have an equivalence in comparison to traditional HF-MRI^73^ or within a defined acceptable clinical tolerance margin (such as 10%) or comparison of outcomes for a large range of neurological conditions and diseases and not lower than HF-MRI (non-inferiority). This study has helped highlight that there is currently pilot-level data from studies. Still, there is an evidence gap of missing large-scale, large-sample, multicentre clinical trials applying standardised observer performance interpretation methodology. Although outcome measures such as non-inferiority endpoints are often applied in clinical trials to assess whether a new intervention (ULF-MRI) is not unacceptably worse than an existing intervention (in this case HF-MRI) through primary outcomes (survival, symptom reduction, medical response, etc), there is at present insufficient data to evidence this. However, there are current studies attempting to apply AI to improve ULF-MRI image quality to that of near HF-MRI.^43 74–77^

There are overlaps in level one^33 35^ evidence in terms of volumetric outcome metrics, especially around the use of dementia/atrophy modelling to improve the image resolution and quality of ULF-MRI through deep learning^76 78 79^ and AI image quality processing.^43 74–77^ But an over-reliance on technical performance^33 35^ alone will not be sufficient for the clinical upscaling and roll-out of ULF-MRI, or to ensure improvements in high-level^33 35^ patient care, or to guarantee clinical benefits or value. Studies reporting high diagnostic accuracy in lesion/disease detection need to provide further evidence, in radiological terms, that changes in patient management improve outcomes. This is often difficult to establish, placing the onus solely on imaging rather than on clinician experience, training, shared decision-making with other diagnostic tests and strict guidelines. Thus, clinical trial research to address higher levels of diagnostic thinking, patient outcomes and societal efficacy will be required to support investment in the clinical adoption of ULF-MRI, which translates directly into faster, more cost-effective patient access to MRI imaging and diagnoses, benefiting clinical decisions and care pathways, beyond studies on discussions^80–87^ of the cost-effectiveness^24 26 88^ and safety^89 90^ of using ULF-MRI for neurological imaging.

### Strengths and limitations

The study followed the PRISMA guidelines^49^ and assessed risk of bias through the QUADAS-3 tool.^55^ Heterogeneity and risk of bias across studies reflect the substantial variation in study designs and environments, patient populations, pathologies, lesion size and conspicuity, comparator reference tests (HF-MRI and CT), imaging protocols and study design, which may have contributed to imprecision and potential overestimation of diagnostic performance accuracy. Therefore, pooled estimates should be interpreted with caution given the methodological heterogeneity and variable study quality. Additionally, bivariate random-effects model or hierarchical summary ROC was not conducted. We recommend that future reviews consider a formal certainty-of-evidence evaluation to support interpretation of diagnostic accuracy estimates. This study only assessed the level two Mackenzie and Dixon^35^ and Fryback and Thornbury^33^ diagnostic performance levels and not the technical performance (image quality) or impact on treatment, management or wider health outcomes of the patients scanned.

## Conclusion

In this review, we have presented the current evidence on the diagnostic accuracy of ULF-MRI in neurological examinations. There were n=17 ULF-MRI studies identified, with a summary diagnostic performance of 90% sensitivity (95% CI 0.82 to 0.94), 98% specificity (95% CI 0.97 to 0.99) and an AUC of 0.99. The significant heterogeneity (χ^2^=39.77; p=0.008; I^2^=59.8%) in study methodologies (pilot to observational cohorts, random and convenience recruitment, patient sample sizes, range or limitations of pathological conditions imaged, blinding and unblinded to a mix of comparator imaging and the number of observers reporting the imaging) warrants caution when interpreting the meta-analysis results.

The studies demonstrated high diagnostic accuracy for gross structural neurological conditions and strong specificity, but sensitivity for small and subtle lesions below 6 mm was limited with ULF-MRI. However, ULF-MRI is not positioned within the studies as a replacement for conventional HF-MRI (1.5T/3T), but more as a complementary monitoring, triage and imaging follow-up at bedside assessment, particularly for critical patients.

## Supplementary material

10.1136/bmjopen-2026-124306 online supplemental file 1

10.1136/bmjopen-2026-124306 online supplemental file 2

## Data Availability

Data are available upon reasonable request.
