# Supplementary material for: Diagnostic performance of ultra-low-field neurological magnetic resonance imaging: a systematic review and meta-analysis
Source: BMJ Open. 2026 Oct 30;16(10):e124306. doi: 10.1136/bmjopen-2026-124306 (PMC13723857; doi:10.1136/bmjopen-2026-124306)
Supplement: Supplementary file 1 [file bmjopen-2026-124306.reviewer_comments.pdf]

PEER REVIEW HISTORY

BMJ Open publishes all reviews undertaken for accepted manuscripts. Reviewers are asked to complete a checklist review form and are provided with free text boxes to elaborate on their assessment. These free text comments are reproduced below.

ARTICLE DETAILS

Title (Provisional)

Diagnostic performance of ultra-low field neurological magnetic resonance imaging: a systematic review and meta-analysis

Authors

Lockwood, Paul; Barrett, Elizabeth

VERSION 1 - REVIEW

**Reviewer**	**1**
**Name**	**Dack, Kyle**
**Affiliation**	**University of Bristol, MRC Integrative Epidemiology Unit**
**Date**	**01-Aug-2026**
**COI**	**None**

Statistical review summary:

The paper is presented well and adheres in the most part with PRISMA reporting guidelines. However, the statistical methodology could be described in more detail. There are some limitations in the evidence base - moderate heterogeneity, variable RoB, and the impact of these on the certainty of the results could also be explored in more detail.

Abstract:

1. The abstract is clear and contains all important elements - no suggestions.

Methods:

2. Search terms: The overall approach is presented, but the specific query will vary between databases due to differences in terms, options, etc. It is recommended in PRISMA to share the database-specific queries to strengthen the replicability, e.g. in a supplementary file.

3. Please clarify in the statistical analysis how the meta-analyses were performed. The software and the use of forest plots is reported, but not the statistical model.

4. It is difficult to fully assess due to the prior comment, but current methodological guidance such as the Cochrane handbook recommend hierarchical models which take into account the relationship between sensitivity and specificity. For example bivariate random-

effects or HSROC. Please clarify if these were applied, or the rationale for using alternative methods
https://methods.cochrane.org/sites/methods.cochrane.org.sdt/files/uploads/Chapter%2010%20-%20Version%201.0.pdf

5. Heterogeneity was quantified but not explored or interpreted. There was moderate heterogeneity identified for certain estimates, indicating there could be variation in the true accuracy based on certain factors. The study numbers may have made subgroup analyses not feasible, but this should be communicated to readers. Heterogeneity could still be explored narratively and it would strengthen the subsequent interpretation. For example in figure 3 there appear three major outliers (Bauer 2024, Lim 2024, von Danwitz 2025). Are there any commonalities which distinguish these from the other studies?

Whether or not it can be explained I suggest this could also be evaluated more prominently in the discussion. Currently it is presented as a limitation but it may have a significant impact on the interpretation.

6. Certainty of evidence: There is not currently a systematic evaluation of the certainty of evidence. While this is not a requirement in PRISMA for meta-analyses of DTA, there are adaptations of GRADE available (https://pmc.ncbi.nlm.nih.gov/articles/PMC2386626/). The authors could consider whether it is suitable to apply GRADE, as it may give more clarity to how certain the results can be interpreted.

Results:

7. " Characteristics of included studies" in the third line I don't think "although" is necessary.

8. Meta-analysis results - I recommend including the 95% CI with the point estimate, this is currently reported in the figure only.

9. " However, there was some uncertainty in the findings ($\chi^2 = 23.88$; $p = 0.004$; $I^2 = 62.3\%$) due to moderate to substantial variance (heterogeneity) between studies (sample sizes, study designs and methods, conditions, etc.);" I think this could be made more precise. The findings indicate there is moderate heterogeneity in the PLR across studies, which could reflect differences in performance across study designs, settings, or populations. However, with 10 studies it should be feasible to explore what patterns of heterogeneity there really are which could explain the variation.

Discussion:

10. The interpretation is reasonable but could be strengthened by further comment on the limitations or sources of uncertainty in the study. There was moderate heterogeneity which has some impact on the interpretation, plus variable RoB and sample size. The current limitations are quite brief and could explore the implication of these elements in more detail.

**Reviewer**	**2**
**Name**	**Faraoni, David**
**Affiliation**	**Boston Children's Hospital**
**Date**	**17-Aug-2026**
**COI**	**None**

In this systematic review, the authors summarized diagnostic accuracy of ultra-field MRI in neurosurgical imaging. They review the existing literature and were able to include 17 articles. The authors should be congratulated for their well designed and well executed review of the literature.

1. Although the authors are reviewing an important topic, I am not sure the authors appropriately described the study objectives and hypothesis. I am also not convinced the literature includes enough high quality publications to answer the research question in a meaningful way. The authors leave the readers with more questions than answers.

2. On page 1 (Abstract), specificity is listed as 93% (95% CI, 0.93-0.98). A lower confidence limit equal to the point estimate (0.93) is likely a typo.

3. On page 9 (Results), the manuscript states: "ranging from n=860 to n=189...". Reviewing Table 2 indicates this was intended to read n=8?

4. High statistical heterogeneity is present in sensitivity (I =59.8%) and negative likelihood ratios (I =76.5%). While the text cautions the reader, the discussion would benefit from explicit subgroup explorations (e.g., adult vs. pediatric/neonatal, or specific pathology types like acute stroke vs. MS) to explain sources of heterogeneity.

5. 8 of the 17 studies used a mix of CT and MRI as comparator reference standards. Because CT and MRI have significantly different sensitivity profiles for early ischemic changes or subtle demyelinating lesions, the authors should further elaborate on how this mixing impacts the risk of verification bias.

VERSION 1 - AUTHOR RESPONSE

Reviewer: 1
Dr. Kyle Dack, University of Bristol
Comments to the Authors:

Authors- *Thank you for the peer review, and we appreciate the support provided by these comments in helping with our submission to the journal and disseminating this research to the wider international readership of the journal.*

Statistical review summary:
The paper is presented well and adheres in the most part with PRISMA reporting guidelines. However, the statistical methodology could be described in more detail. There are some limitations in the evidence base - moderate heterogeneity, variable RoB, and the impact of these on the certainty of the results could also be explored in more detail.

Authors- *Thank you we have now expanded upon the Heterogeneity narratively as requested including the risk of bias such as CT as a reference standard compared to MRI, sample size, lesion size, pathologies, image protocols, flow and timing in the discussion section and the limitations section.*

Abstract:
1. The abstract is clear and contains all important elements - no suggestions.

Authors- *Thank you*

Methods:
2. Search terms: The overall approach is presented, but the specific query will vary between databases due to differences in terms, options, etc. It is recommended in PRISMA to share the database-specific queries to strengthen the replicability, e.g. in a supplementary file.

Authors- *Thank you this change has now been actioned in the submitted supplementary materials 1 documents now.*

3. Please clarify in the statistical analysis how the meta-analyses were performed. The software and the use of forest plots is reported, but not the statistical model.

Authors- *Thank you this change has now been actioned in the methods section.*

4. It is difficult to fully assess due to the prior comment, but current methodological guidance such as the Cochrane handbook recommend hierarchical models which take into account the relationship between sensitivity and specificity. For example bivariate random-effects or HSROC. Please clarify if these were applied, or the rationale for using alternative methods https://methods.cochrane.org/sites/methods.cochrane.org.sdt/files/uploads/Chapter%2010%20-%20Version%201.0.pdf

Authors- *Thank you, we acknowledge that Cochrane recommends hierarchical approaches such as the bivariate random-effects model or HSROC model for meta-analysis of diagnostic test accuracy studies, and whilst some authors will do this to adhere to Cochrane*

submission (this paper is not being submitted to Cochrane). However, the analysis was performed using the available Meta-DiSc software, which supports SROC analysis, you are right it does not implement hierarchical modelling. Consequently, a SROC approach was used to synthesise overall diagnostic performance due to the software used and with access to. Whilst hierarchical models may provide estimates by jointly modelling sensitivity and specificity, (we don't have a licence for STRATA) SROC analysis still remains an established and widely reported method in diagnostic test accuracy reviews. This limitation has been acknowledged in the manuscript.

5. Heterogeneity was quantified but not explored or interpreted. There was moderate heterogeneity identified for certain estimates, indicating there could be variation in the true accuracy based on certain factors. The study numbers may have made subgroup analyses not feasible, but this should be communicated to readers. Heterogeneity could still be explored narratively and it would strengthen the subsequent interpretation. For example in figure 3 there appear three major outliers (Bauer 2024, Lim 2024, von Danwitz 2025). Are there any commonalities which distinguish these from the other studies?

Authors-*Thank you we have now expanded upon the Heterogeneity narratively as requested including the risk of bias such as CT as a reference standard compared to MRI, sample size, lesion size, pathologies, image protocols, flow and timing in the discussion section and the limitations section.*

Whether or not it can be explained I suggest this could also be evaluated more prominently in the discussion. Currently it is presented as a limitation but it may have a significant impact on the interpretation.

Authors- *Thank you we have now expanded upon the Heterogeneity narratively as requested in the discussion section and the limitations section.*

6. Certainty of evidence: There is not currently a systematic evaluation of the certainty of evidence. While this is not a requirement in PRISMA for meta-analyses of DTA, there are adaptations of GRADE available (https://pmc.ncbi.nlm.nih.gov/articles/PMC2386626/). The authors could consider whether it is suitable to apply GRADE, as it may give more clarity to how certain the results can be interpreted.

Authors- *Thank you we appreciate the reviewer's suggestion, while adapted GRADE approaches for DTA reviews are used in other studies and may provide additional insight into the certainty of evidence, this assessment was not planned as part of the original review methodology. As PRISMA guidance does not require a formal certainty-of-evidence evaluation for DTA meta-analyses, we do not believe its omission compromises the validity of the review. Implementing GRADE at this stage would require substantial re-analysis and extensive revision of the manuscript, including major additions to the Methods, Results, and Discussion sections. Given the journal's word-count restrictions and the scope of the current revision, we have not undertaken a GRADE assessment. We have, however, noted this as a limitation and recommend that future reviews consider a formal certainty-of-evidence evaluation to support interpretation of diagnostic accuracy estimates.*

Results:
7. " Characteristics of included studies" in the third line I don’t think "although" is necessary.

Authors- *Thank you this has been rephrased.*

8. Meta-analysis results - I recommend including the 95% CI with the point estimate, this is currently reported in the figure only.

Authors- *Thank you this has been added to the manuscript.*

9. " However, there was some uncertainty in the findings ($\chi^2 = 23.88$; $p = 0.004$; $I^2 = 62.3\%$) due to moderate to substantial variance (heterogeneity) between studies (sample sizes, study designs and methods, conditions, etc.);" I think this could be made more precise. The findings indicate there is moderate heterogeneity in the PLR across studies, which could reflect differences in performance across study designs, settings, or populations. However, with 10 studies it should be feasible to explore what patterns of heterogeneity there really are which could explain the variation.

Authors- *Thank you we have now expanded upon the Heterogeneity narratively as requested including the risk of bias such as CT as a reference standard compared to MRI, sample size, lesion size, pathologies, image protocols, flow and timing in the discussion section and the limitations section.*

Discussion:
10. The interpretation is reasonable but could be strengthened by further comment on the limitations or sources of uncertainty in the study. There was moderate heterogeneity which has some impact on the interpretation, plus variable RoB and sample size. The current limitations are quite brief and could explore the implication of these elements in more detail.

Authors- *Thank you we have now expanded upon the Heterogeneity in the limitations section as advised.*

Authors- *Again, we thank you for your time and comments and hope the revisions will now be accepted for publication.*

Reviewer: 2
Dr. David Faraoni, Boston Children's Hospital
Comments to the Authors:

In this systematic review, the authors summarized diagnostic accuracy of ultra-field MRI in neurosurgical imaging. They review the existing literature and were able to include 17 articles. The authors should be congratulated for their well designed and well executed review of the literature.

Authors- *Thank you for the peer review, and we appreciate the support provided by these comments in helping with our submission to the journal and disseminating this research to the wider international readership of the journal.*

1. Although the authors are reviewing an important topic, I am not sure the authors appropriately described the study objectives and hypothesis. I am also not convinced the literature includes enough high quality publications to answer the research question in a meaningful way. The authors leave the readers with more questions than answers.

Authors- *We thank the reviewer for highlighting these important points. We respectfully disagree that the study objectives were insufficiently described. The primary objective is explicitly stated in both the Abstract and Introduction:*

"To systematically review the reported level of diagnostic performance of Ultra-Low Field Magnetic Resonance Imaging (ULF-MRI) in brain imaging of both paediatric (including neonates and infants) and adult populations across a range of neurological conditions."

Furthermore, the scope of the review is clearly defined:

"This systematic literature review aims to assess the current level two evidence on the diagnostic accuracy of ULF-MRI in detecting neurological conditions and pathologies. The study will not assess the higher Mackenzie and Dixon or Fryback and Thornbury clinical efficacy levels relating to downstream patient management, treatment outcomes, or service delivery benefits."

As this review was designed to evaluate the current evidence base, rather than test a specific intervention effect, we did not formulate a formal hypothesis. Instead, the review sought to systematically identify, appraise, and synthesise the available evidence regarding the diagnostic performance of ULF-MRI. We acknowledge the reviewer's concern regarding the limited number of high-quality studies. Indeed, one of the key findings of the review is that the current evidence base remains relatively small and heterogeneous. However, this limitation itself is an important finding and reflects the emerging nature of ULF-MRI technology. Systematic reviews are valuable not only when evidence is abundant, but also when they identify gaps in knowledge, limitations in study quality, and priorities for future research. Our review therefore provides a comprehensive assessment of the existing literature, highlights areas where evidence is currently insufficient, and helps inform the design of future diagnostic accuracy studies. To address the reviewer's concern, we have clarified in the manuscript that the limited number and quality of available studies restrict the strength of conclusions that can be drawn, and that further well-designed prospective diagnostic accuracy studies are needed before definitive conclusions regarding the clinical role of ULF-MRI can be made.

2. On page 1 (Abstract), specificity is listed as 93% (95% CI, 0.93-0.98). A lower confidence limit equal to the point estimate (0.93) is likely a typo.

Authors- *Yes agreed, this has now been corrected.*

3. On page 9 (Results), the manuscript states: "ranging from n=860 to n=189...". Reviewing

Table 2 indicates this was intended to read n=8?

Authors- *Thank you for raising this point, it may be an issue with the PDF formatting at the journals end the n=860 in the manuscript refers to a sample of n=8[60] (as in the sample of n=8 is from the cited superscript reference 60) but the range does extend to a study of a sample of n=189 in the cited superscript reference 66. In text it reads as follows*

'The selection of studies included a wide sample size of patients' scans ranging from *n*=8[60] to *n*=189,[66] with a mix of neurological pathological conditions detected.'

4. High statistical heterogeneity is present in sensitivity (I =59.8%) and negative likelihood ratios (I =76.5%). While the text cautions the reader, the discussion would benefit from explicit subgroup explorations (e.g., adult vs. pediatric/neonatal, or specific pathology types like acute stroke vs. MS) to explain sources of heterogeneity.

Authors- *Thank you we have now expanded upon the Heterogeneity narratively as requested including the risk of bias such as CT as a reference standard compared to MRI, sample size, lesion size, pathologies, image protocols, flow and timing in the discussion section and the limitations section.*

5. 8 of the 17 studies used a mix of CT and MRI as comparator reference standards. Because CT and MRI have significantly different sensitivity profiles for early ischemic changes or subtle demyelinating lesions, the authors should further elaborate on how this mixing impacts the risk of verification bias.

Authors- *We thank the reviewer for this important observation. We agree that the use of both CT and MRI as comparator reference standards across 8 of the 17 included studies introduces heterogeneity in the assessment of diagnostic performance. MRI is generally considered more sensitive than CT for the detection of subtle neurological abnormalities, including early ischaemic changes, small infarcts, demyelinating lesions, and other parenchymal pathologies. Consequently, studies using CT as the reference standard may underestimate lesion detection compared with studies using MRI, which could influence reported estimates of diagnostic accuracy.*
We would, however, note that this issue is more appropriately considered a limitation of reference standard variability and potential misclassification and/or verification bias within the primary studies. To address this concern, we have expanded the Discussion to acknowledge that the mixed use of CT and MRI reference standards may have influenced the reported diagnostic accuracy of ULF-MRI. We have also clarified in the Risk of Bias assessment that studies employing CT as the reference standard may be at greater risk of reference standard bias because CT has lower sensitivity than conventional MRI for certain neurological pathologies. As a result, the true diagnostic capability of ULF-MRI may have been either underestimated or overestimated depending on the pathology under investigation and the comparator modality used.

Authors- *Again, we thank you for your time and comments and hope the revisions will now be accepted for publication.*

COI statement(s):

Reviewer: 1
If you have selected 'Yes' above, please provide details of any competing interests.: NA.

Authors- *NA*

Reviewer: 2
If you have selected 'Yes' above, please provide details of any competing interests.: Not applicable.

Authors- *NA*

VERSION 2 - REVIEW

**Reviewer**	**1**
**Name**	**Dack, Kyle**
**Affiliation**	**University of Bristol, MRC Integrative Epidemiology Unit**
**Date**	**22-Sep-2026**
**COI**	

The revisions fully addressed my queries and suggestions, including an detailed description of possible sources of heterogeneity. However, this was placed at the start of the discussion, and it is a little jarring to begin this section. The authors and editorial team may wish to consider if this section could be reorganised, so that the discussion begins with a general summary of the key findings.

VERSION 2 - AUTHOR RESPONSE

Reviewer: 1
Dr. Kyle Dack, University of Bristol

Authors - *Thank you for the continued support and peer review, and we appreciate the comments in helping with our submission to the journal and disseminating this research to the wider international readership of the journal*

The revisions fully addressed my queries and suggestions, including an detailed description of possible sources of heterogeneity. However, this was placed at the start of the discussion, and it is a little jarring to begin this section. The authors and editorial team may wish to consider if this section could be reorganised, so that the discussion begins with a general summary of the key findings.

Authors – *Thank you for this helpful suggestion. The Discussion section has now been revised and restructured to align more closely with the recommended format. Specifically, we*

have organised the Discussion to include: (i) a summary of the main findings, (ii) interpretation of the results, including discussion of sources of heterogeneity, (iii) comparison with existing literature, (iv) implications of the findings, (v) strengths and limitations of the study. We believe these revisions have improved the clarity, coherence, and overall flow of the Discussion.

Reviewer: 1
If you have selected 'Yes' above, please provide details of any competing interests.: NA.

Authors – *NA*
