# Supplementary material for: Diagnostic performance of ultra-low-field neurological magnetic resonance imaging: a systematic review and meta-analysis
Source: BMJ Open. 2026 Oct 30;16(10):e124306. doi: 10.1136/bmjopen-2026-124306 (PMC13723857; doi:10.1136/bmjopen-2026-124306)
Supplement: Supplementary file 2 [file bmjopen-16-10-s001.docx]

**Supplementary material 1 - Search strategy databases, input words, filters, and limits**

Database: PubMed	Date accessed: 24.02.2026

| Line | Search Strategy |
| --- | --- |
| #1 | Combination of controlled vocabulary (hierarchical controlled vocabularies for health sciences (e.g., MeSH) and free-text terms: Adult; Brain; Brain disease; Brain infarction; Brain injury; Brain haemorrhage, Brain neoplasms, Brian injuries, Brain oedema, Central Nervous System, Cerebrovascular trauma, Cerebral haemorrhage, Hydrocephalus, Intracranial arterial diseases, Dementia; Stroke, Humans; Magnetic Resonance Imaging; Neuroimaging; Neuroradiography, Patient Outcome Assessment; Point-of-Care Systems; Sensitivity and Specificity, Systematic Reviews as Topic; Workflow |
| #2 | The Boolean terms included in the PubMed Advanced Search Query box: ("MRI" OR "magnetic resonance imaging" OR "ultra low field" OR "ultra-low-field" OR "low-field MRI" OR "portable MRI" OR "0.064T" OR "64mT" OR "0.1T" OR "0.2T" OR "portable MRI" OR "portable scanner" OR "Low-cost MRI" OR "Low-field MRI" OR "low-field brain MRI" OR "Point of Care MRI" OR pMRI OR "POC MRI" OR "Mobile MRI" OR mMRI OR "Mobile point of care MRI" OR "Low Tesla Magnetic resonance imaging" OR "Low strength Magnetic resonance imaging" OR "Low strength MRI" OR "bedside MRI" OR "bedside portable MRI" OR "brain only MRI" OR "head only MRI" OR "HyperfineSwoop" OR Hyperfine OR "Halbach MRI" OR Halbach) |
| #3 | The Boolean terms included in the PubMed Advanced Search Query box: ("Human patient" OR "brain" OR "cerebral" OR "intracranial" OR "neurological" OR "stroke" OR "tumor" OR "tumour" OR "infection" OR "haemorrhage" OR "hemorrhage" OR "TBI" OR "Brain Diseases") |
| #4 | The Boolean terms included in the PubMed Advanced Search Query box: ("Computed Tomography" OR "CT" OR "MRI" OR "magnetic resonance imaging” OR “high field" OR "High-field MRI" OR "1.5T" OR "3T" OR "reference standard imaging" OR "standard neurological imaging") |
| #5 | The Boolean terms included in the PubMed Advanced Search Query box: ("Diagnosis" OR "diagnostic accuracy" OR "sensitivity" OR "specificity" OR "agreement" OR "Positive Predictive Value" OR "Negative Predictive Value" OR "Reciever Operating Characteristic" OR "Area Under the Curve" OR "AuC" OR "Probability Values" OR "p-values" OR "Liklihood ratios" OR "Observer performance" OR "detection") No population terms or condition-specific filters (e.g., treatment outcomes) |
| #6 | #2 AND #3 |
| #7 | #2 AND #3 AND #5 |
| Filters | Eligibility criteria comprised published, peer-reviewed primary data studies without date restriction, involving human subjects of any age or gender undergoing brain imaging, irrespective of health or clinical indications. Studies were required to have used ULF-MRI, to report diagnostic accuracy or image evaluation, and to provide comparative findings with conventional cross-sectional neuroimaging modalities such as MRI, computed tomography (CT), or other relevant clinical reference standards.  No restrictions country of research, study setting, study design, patient population, MRI manufacturer or vendor, or MRI protocol. All relevant studies published in English were included, with no restriction on publication date. |
| Limits | Exclusions of studies focused on non-brain or non-head imaging (e.g., other anatomical regions), phantom studies (physics testing), animal imaging, absence of diagnostic outcome data, or the use of artificial intelligence (AI) software for image reading, interpretation, or quality enhancement |

Database: Medline	Date accessed: 26.02.2026

| Line | Search Strategy |
| --- | --- |
| #1 | Combination of controlled vocabulary (hierarchical controlled vocabularies for health sciences (e.g., MeSH) and free-text terms: Adult; Brain; Brain disease; Brain infarction; Brain injury; Brain haemorrhage, Brain neoplasms, Brian injuries, Brain oedema, Central Nervous System, Cerebrovascular trauma, Cerebral haemorrhage, Hydrocephalus, Intracranial arterial diseases, Dementia; Stroke, Humans; Magnetic Resonance Imaging; Neuroimaging; Neuroradiography, Patient Outcome Assessment; Point-of-Care Systems; Sensitivity and Specificity, Systematic Reviews as Topic; Workflow |
| #2 | The Boolean terms included in the Medline Advanced Search Query box: ("MRI" OR "magnetic resonance imaging" OR "ultra low field" OR "ultra-low-field" OR "low-field MRI" OR "portable MRI" OR "0.064T" OR "64mT" OR "0.1T" OR "0.2T" OR "portable MRI" OR "portable scanner" OR "Low-cost MRI" OR "Low-field MRI" OR "low-field brain MRI" OR "Point of Care MRI" OR pMRI OR "POC MRI" OR "Mobile MRI" OR mMRI OR "Mobile point of care MRI" OR "Low Tesla Magnetic resonance imaging" OR "Low strength Magnetic resonance imaging" OR "Low strength MRI" OR "bedside MRI" OR "bedside portable MRI" OR "brain only MRI" OR "head only MRI" OR "HyperfineSwoop" OR Hyperfine OR "Halbach MRI" OR Halbach) |
| #3 | The Boolean terms included in the Medline Advanced Search Query box:("Human patient" OR "brain" OR "cerebral" OR "intracranial" OR "neurological" OR "stroke" OR "tumor" OR "tumour" OR "infection" OR "haemorrhage" OR "hemorrhage" OR "TBI" OR "Brain Diseases") |
| #4 | The Boolean terms included in the Medline Advanced Search Query box: ("Computed Tomography" OR "CT" OR "MRI" OR "magnetic resonance imaging” OR “high field" OR "High-field MRI" OR "1.5T" OR "3T" OR "reference standard imaging" OR "standard neurological imaging") |
| #5 | The Boolean terms included in the Medline Advanced Search Query box: ("Diagnosis" OR "diagnostic accuracy" OR "sensitivity" OR "specificity" OR "agreement" OR "Positive Predictive Value" OR "Negative Predictive Value" OR "Reciever Operating Characteristic" OR "Area Under the Curve" OR "AuC" OR "Probability Values" OR "p-values" OR "Liklihood ratios" OR "Observer performance" OR "detection") No population terms or condition-specific filters (e.g., treatment outcomes) |
| #6 | #2 AND #3 |
| #7 | #2 AND #3 AND #5 |
| Filters | Eligibility criteria comprised published, peer-reviewed primary data studies without date restriction, involving human subjects of any age or gender undergoing brain imaging, irrespective of health or clinical indications. Studies were required to have used ULF-MRI, to report diagnostic accuracy or image evaluation, and to provide comparative findings with conventional cross-sectional neuroimaging modalities such as MRI, computed tomography (CT), or other relevant clinical reference standards.  No restrictions country of research, study setting, study design, patient population, MRI manufacturer or vendor, or MRI protocol. All relevant studies published in English were included, with no restriction on publication date. |
| Limits | Exclusions of studies focused on non-brain or non-head imaging (e.g., other anatomical regions), phantom studies (physics testing), animal imaging, absence of diagnostic outcome data, or the use of artificial intelligence (AI) software for image reading, interpretation, or quality enhancement |

CINHAL Central (EBSCO)	Date accessed: 27.02.2026

| Line | Search Strategy |
| --- | --- |
| #1 | Combination of controlled vocabulary (hierarchical controlled vocabularies for health sciences (e.g., MeSH) and free-text terms: Adult; Brain; Brain disease; Brain infarction; Brain injury; Brain haemorrhage, Brain neoplasms, Brian injuries, Brain oedema, Central Nervous System, Cerebrovascular trauma, Cerebral haemorrhage, Hydrocephalus, Intracranial arterial diseases, Dementia; Stroke, Humans; Magnetic Resonance Imaging; Neuroimaging; Neuroradiography, Patient Outcome Assessment; Point-of-Care Systems; Sensitivity and Specificity, Systematic Reviews as Topic; Workflow |
| #2 | The Boolean terms included in the CINHAL Advanced Search Query box: ("MRI" OR "magnetic resonance imaging" OR "ultra low field" OR "ultra-low-field" OR "low-field MRI" OR "portable MRI" OR "0.064T" OR "64mT" OR "0.1T" OR "0.2T" OR "portable MRI" OR "portable scanner" OR "Low-cost MRI" OR "Low-field MRI" OR "low-field brain MRI" OR "Point of Care MRI" OR pMRI OR "POC MRI" OR "Mobile MRI" OR mMRI OR "Mobile point of care MRI" OR "Low Tesla Magnetic resonance imaging" OR "Low strength Magnetic resonance imaging" OR "Low strength MRI" OR "bedside MRI" OR "bedside portable MRI" OR "brain only MRI" OR "head only MRI" OR "HyperfineSwoop" OR Hyperfine OR "Halbach MRI" OR Halbach) |
| #3 | The Boolean terms included in the CINHAL Advanced Search Query box: ("Human patient" OR "brain" OR "cerebral" OR "intracranial" OR "neurological" OR "stroke" OR "tumor" OR "tumour" OR "infection" OR "haemorrhage" OR "hemorrhage" OR "TBI" OR "Brain Diseases") |
| #4 | The Boolean terms included in the CINHAL Advanced Search Query box: ("Computed Tomography" OR "CT" OR "MRI" OR "magnetic resonance imaging” OR “high field" OR "High-field MRI" OR "1.5T" OR "3T" OR "reference standard imaging" OR "standard neurological imaging") |
| #5 | The Boolean terms included in the CINHAL Advanced Search Query box: ("Diagnosis" OR "diagnostic accuracy" OR "sensitivity" OR "specificity" OR "agreement" OR "Positive Predictive Value" OR "Negative Predictive Value" OR "Reciever Operating Characteristic" OR "Area Under the Curve" OR "AuC" OR "Probability Values" OR "p-values" OR "Liklihood ratios" OR "Observer performance" OR "detection") No population terms or condition-specific filters (e.g., treatment outcomes) |
| #6 | #2 AND #3 |
| #7 | #2 AND #3 AND #5 |
| Filters | Eligibility criteria comprised published, peer-reviewed primary data studies without date restriction, involving human subjects of any age or gender undergoing brain imaging, irrespective of health or clinical indications. Studies were required to have used ULF-MRI, to report diagnostic accuracy or image evaluation, and to provide comparative findings with conventional cross-sectional neuroimaging modalities such as MRI, computed tomography (CT), or other relevant clinical reference standards.  No restrictions country of research, study setting, study design, patient population, MRI manufacturer or vendor, or MRI protocol. All relevant studies published in English were included, with no restriction on publication date. |
| Limits | Exclusions of studies focused on non-brain or non-head imaging (e.g., other anatomical regions), phantom studies (physics testing), animal imaging, absence of diagnostic outcome data, or the use of artificial intelligence (AI) software for image reading, interpretation, or quality enhancement |

Embase (OVID)	Date accessed: 03.03.2026

| Line | Search Strategy |
| --- | --- |
| #1 | Combination of controlled vocabulary (hierarchical controlled vocabularies for health sciences (e.g., MeSH) and free-text terms: Adult; Brain; Brain disease; Brain infarction; Brain injury; Brain haemorrhage, Brain neoplasms, Brian injuries, Brain oedema, Central Nervous System, Cerebrovascular trauma, Cerebral haemorrhage, Hydrocephalus, Intracranial arterial diseases, Dementia; Stroke, Humans; Magnetic Resonance Imaging; Neuroimaging; Neuroradiography, Patient Outcome Assessment; Point-of-Care Systems; Sensitivity and Specificity, Systematic Reviews as Topic; Workflow |
| #2 | The Boolean terms included in the EMBASE Multi-Search Field Query box: ("MRI" OR "magnetic resonance imaging" OR "ultra low field" OR "ultra-low-field" OR "low-field MRI" OR "portable MRI" OR "0.064T" OR "64mT" OR "0.1T" OR "0.2T" OR "portable MRI" OR "portable scanner" OR "Low-cost MRI" OR "Low-field MRI" OR "low-field brain MRI" OR "Point of Care MRI" OR pMRI OR "POC MRI" OR "Mobile MRI" OR mMRI OR "Mobile point of care MRI" OR "Low Tesla Magnetic resonance imaging" OR "Low strength Magnetic resonance imaging" OR "Low strength MRI" OR "bedside MRI" OR "bedside portable MRI" OR "brain only MRI" OR "head only MRI" OR "HyperfineSwoop" OR Hyperfine OR "Halbach MRI" OR Halbach) |
| #3 | The Boolean terms included in the EMBASE Multi-Search Field Query box: ("Human patient" OR "brain" OR "cerebral" OR "intracranial" OR "neurological" OR "stroke" OR "tumor" OR "tumour" OR "infection" OR "haemorrhage" OR "hemorrhage" OR "TBI" OR "Brain Diseases") |
| #4 | The Boolean terms included in the EMBASE Multi-Search Field Query box: ("Computed Tomography" OR "CT" OR "MRI" OR "magnetic resonance imaging” OR “high field" OR "High-field MRI" OR "1.5T" OR "3T" OR "reference standard imaging" OR "standard neurological imaging") |
| #5 | The Boolean terms included in the EMBASE Multi-Search Field Query box: ("Diagnosis" OR "diagnostic accuracy" OR "sensitivity" OR "specificity" OR "agreement" OR "Positive Predictive Value" OR "Negative Predictive Value" OR "Reciever Operating Characteristic" OR "Area Under the Curve" OR "AuC" OR "Probability Values" OR "p-values" OR "Liklihood ratios" OR "Observer performance" OR "detection") No population terms or condition-specific filters (e.g., treatment outcomes) |
| #6 | #2 AND #3 |
| #7 | #2 AND #3 AND #5 |
| Filters | Eligibility criteria comprised published, peer-reviewed primary data studies without date restriction, involving human subjects of any age or gender undergoing brain imaging, irrespective of health or clinical indications. Studies were required to have used ULF-MRI, to report diagnostic accuracy or image evaluation, and to provide comparative findings with conventional cross-sectional neuroimaging modalities such as MRI, computed tomography (CT), or other relevant clinical reference standards.  No restrictions country of research, study setting, study design, patient population, MRI manufacturer or vendor, or MRI protocol. All relevant studies published in English were included, with no restriction on publication date. |
| Limits | Exclusions of studies focused on non-brain or non-head imaging (e.g., other anatomical regions), phantom studies (physics testing), animal imaging, absence of diagnostic outcome data, or the use of artificial intelligence (AI) software for image reading, interpretation, or quality enhancement |

Web of Science	Date accessed: 10.03.2026

| Line | Search Strategy |
| --- | --- |
| #1 | Combination of controlled vocabulary (hierarchical controlled vocabularies for health sciences (e.g., MeSH) and free-text terms: Adult; Brain; Brain disease; Brain infarction; Brain injury; Brain haemorrhage, Brain neoplasms, Brian injuries, Brain oedema, Central Nervous System, Cerebrovascular trauma, Cerebral haemorrhage, Hydrocephalus, Intracranial arterial diseases, Dementia; Stroke, Humans; Magnetic Resonance Imaging; Neuroimaging; Neuroradiography, Patient Outcome Assessment; Point-of-Care Systems; Sensitivity and Specificity, Systematic Reviews as Topic; Workflow |
| #2 | The Boolean terms included in the Web of Science Field Search Query box: ("MRI" OR "magnetic resonance imaging" OR "ultra low field" OR "ultra-low-field" OR "low-field MRI" OR "portable MRI" OR "0.064T" OR "64mT" OR "0.1T" OR "0.2T" OR "portable MRI" OR "portable scanner" OR "Low-cost MRI" OR "Low-field MRI" OR "low-field brain MRI" OR "Point of Care MRI" OR pMRI OR "POC MRI" OR "Mobile MRI" OR mMRI OR "Mobile point of care MRI" OR "Low Tesla Magnetic resonance imaging" OR "Low strength Magnetic resonance imaging" OR "Low strength MRI" OR "bedside MRI" OR "bedside portable MRI" OR "brain only MRI" OR "head only MRI" OR "HyperfineSwoop" OR Hyperfine OR "Halbach MRI" OR Halbach) |
| #3 | The Boolean terms included in the Web of Science Field Search Query box: ("Human patient" OR "brain" OR "cerebral" OR "intracranial" OR "neurological" OR "stroke" OR "tumor" OR "tumour" OR "infection" OR "haemorrhage" OR "hemorrhage" OR "TBI" OR "Brain Diseases") |
| #4 | The Boolean terms included in the Web of Science Field Search Query box: ("Computed Tomography" OR "CT" OR "MRI" OR "magnetic resonance imaging” OR “high field" OR "High-field MRI" OR "1.5T" OR "3T" OR "reference standard imaging" OR "standard neurological imaging") |
| #5 | The Boolean terms included in the Web of Science Field Search Query box: ("Diagnosis" OR "diagnostic accuracy" OR "sensitivity" OR "specificity" OR "agreement" OR "Positive Predictive Value" OR "Negative Predictive Value" OR "Reciever Operating Characteristic" OR "Area Under the Curve" OR "AuC" OR "Probability Values" OR "p-values" OR "Liklihood ratios" OR "Observer performance" OR "detection") No population terms or condition-specific filters (e.g., treatment outcomes) |
| #6 | #2 AND #3 |
| #7 | #2 AND #3 AND #5 |
| Filters | Eligibility criteria comprised published, peer-reviewed primary data studies without date restriction, involving human subjects of any age or gender undergoing brain imaging, irrespective of health or clinical indications. Studies were required to have used ULF-MRI, to report diagnostic accuracy or image evaluation, and to provide comparative findings with conventional cross-sectional neuroimaging modalities such as MRI, computed tomography (CT), or other relevant clinical reference standards.  No restrictions country of research, study setting, study design, patient population, MRI manufacturer or vendor, or MRI protocol. All relevant studies published in English were included, with no restriction on publication date. |
| Limits | Exclusions of studies focused on non-brain or non-head imaging (e.g., other anatomical regions), phantom studies (physics testing), animal imaging, absence of diagnostic outcome data, or the use of artificial intelligence (AI) software for image reading, interpretation, or quality enhancement |

Scopus (Elsevier)	Date accessed: 12.03.2026

| Line | Search Strategy |
| --- | --- |
| #1 | Combination of controlled vocabulary (hierarchical controlled vocabularies for health sciences (e.g., MeSH) and free-text terms: Adult; Brain; Brain disease; Brain infarction; Brain injury; Brain haemorrhage, Brain neoplasms, Brian injuries, Brain oedema, Central Nervous System, Cerebrovascular trauma, Cerebral haemorrhage, Hydrocephalus, Intracranial arterial diseases, Dementia; Stroke, Humans; Magnetic Resonance Imaging; Neuroimaging; Neuroradiography, Patient Outcome Assessment; Point-of-Care Systems; Sensitivity and Specificity, Systematic Reviews as Topic; Workflow |
| #2 | The Boolean terms included in the Scopus documents Search Query box: ("MRI" OR "magnetic resonance imaging" OR "ultra low field" OR "ultra-low-field" OR "low-field MRI" OR "portable MRI" OR "0.064T" OR "64mT" OR "0.1T" OR "0.2T" OR "portable MRI" OR "portable scanner" OR "Low-cost MRI" OR "Low-field MRI" OR "low-field brain MRI" OR "Point of Care MRI" OR pMRI OR "POC MRI" OR "Mobile MRI" OR mMRI OR "Mobile point of care MRI" OR "Low Tesla Magnetic resonance imaging" OR "Low strength Magnetic resonance imaging" OR "Low strength MRI" OR "bedside MRI" OR "bedside portable MRI" OR "brain only MRI" OR "head only MRI" OR "HyperfineSwoop" OR Hyperfine OR "Halbach MRI" OR Halbach) |
| #3 | The Boolean terms included in the Scopus documents Search Query box: ("Human patient" OR "brain" OR "cerebral" OR "intracranial" OR "neurological" OR "stroke" OR "tumor" OR "tumour" OR "infection" OR "haemorrhage" OR "hemorrhage" OR "TBI" OR "Brain Diseases") |
| #4 | The Boolean terms included in the Scopus documents Search Query box: ("Computed Tomography" OR "CT" OR "MRI" OR "magnetic resonance imaging” OR “high field" OR "High-field MRI" OR "1.5T" OR "3T" OR "reference standard imaging" OR "standard neurological imaging") |
| #5 | The Boolean terms included in the Scopus documents Search Query box: ("Diagnosis" OR "diagnostic accuracy" OR "sensitivity" OR "specificity" OR "agreement" OR "Positive Predictive Value" OR "Negative Predictive Value" OR "Reciever Operating Characteristic" OR "Area Under the Curve" OR "AuC" OR "Probability Values" OR "p-values" OR "Liklihood ratios" OR "Observer performance" OR "detection") No population terms or condition-specific filters (e.g., treatment outcomes) |
| #6 | #2 AND #3 |
| #7 | #2 AND #3 AND #5 |
| Filters | Eligibility criteria comprised published, peer-reviewed primary data studies without date restriction, involving human subjects of any age or gender undergoing brain imaging, irrespective of health or clinical indications. Studies were required to have used ULF-MRI, to report diagnostic accuracy or image evaluation, and to provide comparative findings with conventional cross-sectional neuroimaging modalities such as MRI, computed tomography (CT), or other relevant clinical reference standards.  No restrictions country of research, study setting, study design, patient population, MRI manufacturer or vendor, or MRI protocol. All relevant studies published in English were included, with no restriction on publication date. |
| Limits | Exclusions of studies focused on non-brain or non-head imaging (e.g., other anatomical regions), phantom studies (physics testing), animal imaging, absence of diagnostic outcome data, or the use of artificial intelligence (AI) software for image reading, interpretation, or quality enhancement |

CENTRAL (Cochrane Central Register of Controlled Trials) Date accessed: 16.03.2026

| Line | Search Strategy |
| --- | --- |
| #1 | Combination of controlled vocabulary (hierarchical controlled vocabularies for health sciences (e.g., MeSH) and free-text terms: Adult; Brain; Brain disease; Brain infarction; Brain injury; Brain haemorrhage, Brain neoplasms, Brian injuries, Brain oedema, Central Nervous System, Cerebrovascular trauma, Cerebral haemorrhage, Hydrocephalus, Intracranial arterial diseases, Dementia; Stroke, Humans; Magnetic Resonance Imaging; Neuroimaging; Neuroradiography, Patient Outcome Assessment; Point-of-Care Systems; Sensitivity and Specificity, Systematic Reviews as Topic; Workflow |
| #2 | The Boolean terms included in the Cochrane Reviews All Text Search Query box: ("MRI" OR "magnetic resonance imaging" OR "ultra low field" OR "ultra-low-field" OR "low-field MRI" OR "portable MRI" OR "0.064T" OR "64mT" OR "0.1T" OR "0.2T" OR "portable MRI" OR "portable scanner" OR "Low-cost MRI" OR "Low-field MRI" OR "low-field brain MRI" OR "Point of Care MRI" OR pMRI OR "POC MRI" OR "Mobile MRI" OR mMRI OR "Mobile point of care MRI" OR "Low Tesla Magnetic resonance imaging" OR "Low strength Magnetic resonance imaging" OR "Low strength MRI" OR "bedside MRI" OR "bedside portable MRI" OR "brain only MRI" OR "head only MRI" OR "HyperfineSwoop" OR Hyperfine OR "Halbach MRI" OR Halbach) |
| #3 | The Boolean terms included in the Cochrane Reviews All Text Search Query box: ("Human patient" OR "brain" OR "cerebral" OR "intracranial" OR "neurological" OR "stroke" OR "tumor" OR "tumour" OR "infection" OR "haemorrhage" OR "hemorrhage" OR "TBI" OR "Brain Diseases") |
| #4 | The Boolean terms included in the Cochrane Reviews All Text Search Query box: ("Computed Tomography" OR "CT" OR "MRI" OR "magnetic resonance imaging” OR “high field" OR "High-field MRI" OR "1.5T" OR "3T" OR "reference standard imaging" OR "standard neurological imaging") |
| #5 | The Boolean terms included in the Cochrane Reviews All Text Search Query box: ("Diagnosis" OR "diagnostic accuracy" OR "sensitivity" OR "specificity" OR "agreement" OR "Positive Predictive Value" OR "Negative Predictive Value" OR "Reciever Operating Characteristic" OR "Area Under the Curve" OR "AuC" OR "Probability Values" OR "p-values" OR "Liklihood ratios" OR "Observer performance" OR "detection") No population terms or condition-specific filters (e.g., treatment outcomes) |
| #6 | #2 AND #3 |
| #7 | #2 AND #3 AND #5 |
| Filters | Eligibility criteria comprised published, peer-reviewed primary data studies without date restriction, involving human subjects of any age or gender undergoing brain imaging, irrespective of health or clinical indications. Studies were required to have used ULF-MRI, to report diagnostic accuracy or image evaluation, and to provide comparative findings with conventional cross-sectional neuroimaging modalities such as MRI, computed tomography (CT), or other relevant clinical reference standards.  No restrictions country of research, study setting, study design, patient population, MRI manufacturer or vendor, or MRI protocol. All relevant studies published in English were included, with no restriction on publication date. |
| Limits | Exclusions of studies focused on non-brain or non-head imaging (e.g., other anatomical regions), phantom studies (physics testing), animal imaging, absence of diagnostic outcome data, or the use of artificial intelligence (AI) software for image reading, interpretation, or quality enhancement |

Google Scholar	Date accessed: 18.03.2026

| Line | Search Strategy |
| --- | --- |
| #1 | Combination of controlled vocabulary (hierarchical controlled vocabularies for health sciences (e.g., MeSH) and free-text terms: Adult; Brain; Brain disease; Brain infarction; Brain injury; Brain haemorrhage, Brain neoplasms, Brian injuries, Brain oedema, Central Nervous System, Cerebrovascular trauma, Cerebral haemorrhage, Hydrocephalus, Intracranial arterial diseases, Dementia; Stroke, Humans; Magnetic Resonance Imaging; Neuroimaging; Neuroradiography, Patient Outcome Assessment; Point-of-Care Systems; Sensitivity and Specificity, Systematic Reviews as Topic; Workflow |
| #2 | The Boolean terms included in the Google Scholar Search Query box: ("MRI" OR "magnetic resonance imaging" OR "ultra low field" OR "ultra-low-field" OR "low-field MRI" OR "portable MRI" OR "0.064T" OR "64mT" OR "0.1T" OR "0.2T" OR "portable MRI" OR "portable scanner" OR "Low-cost MRI" OR "Low-field MRI" OR "low-field brain MRI" OR "Point of Care MRI" OR pMRI OR "POC MRI" OR "Mobile MRI" OR mMRI OR "Mobile point of care MRI" OR "Low Tesla Magnetic resonance imaging" OR "Low strength Magnetic resonance imaging" OR "Low strength MRI" OR "bedside MRI" OR "bedside portable MRI" OR "brain only MRI" OR "head only MRI" OR "HyperfineSwoop" OR Hyperfine OR "Halbach MRI" OR Halbach) |
| #3 | The Boolean terms included in the Google Scholar Search Query box: ("Human patient" OR "brain" OR "cerebral" OR "intracranial" OR "neurological" OR "stroke" OR "tumor" OR "tumour" OR "infection" OR "haemorrhage" OR "hemorrhage" OR "TBI" OR "Brain Diseases") |
| #4 | The Boolean terms included in the Google Scholar Search Query box: ("Computed Tomography" OR "CT" OR "MRI" OR "magnetic resonance imaging” OR “high field" OR "High-field MRI" OR "1.5T" OR "3T" OR "reference standard imaging" OR "standard neurological imaging") |
| #5 | The Boolean terms included in the Google Scholar Search Query box: ("Diagnosis" OR "diagnostic accuracy" OR "sensitivity" OR "specificity" OR "agreement" OR "Positive Predictive Value" OR "Negative Predictive Value" OR "Reciever Operating Characteristic" OR "Area Under the Curve" OR "AuC" OR "Probability Values" OR "p-values" OR "Liklihood ratios" OR "Observer performance" OR "detection") No population terms or condition-specific filters (e.g., treatment outcomes) |
| #6 | #2 AND #3 |
| #7 | #2 AND #3 AND #5 |
| Filters | Eligibility criteria comprised published, peer-reviewed primary data studies without date restriction, involving human subjects of any age or gender undergoing brain imaging, irrespective of health or clinical indications. Studies were required to have used ULF-MRI, to report diagnostic accuracy or image evaluation, and to provide comparative findings with conventional cross-sectional neuroimaging modalities such as MRI, computed tomography (CT), or other relevant clinical reference standards.  No restrictions country of research, study setting, study design, patient population, MRI manufacturer or vendor, or MRI protocol. All relevant studies published in English were included, with no restriction on publication date. |
| Limits | Exclusions of studies focused on non-brain or non-head imaging (e.g., other anatomical regions), phantom studies (physics testing), animal imaging, absence of diagnostic outcome data, or the use of artificial intelligence (AI) software for image reading, interpretation, or quality enhancement |
