# Supplementary material for: Diagnostic performance of ultra-low-field neurological magnetic resonance imaging: a systematic review and meta-analysis
Source: BMJ Open. 2026 Oct 30;16(10):e124306. doi: 10.1136/bmjopen-2026-124306 (PMC13723857; doi:10.1136/bmjopen-2026-124306)
Supplement: Supplementary file 3 [file bmjopen-16-10-s002.docx]

**Supplementary material 2.** Quadas-3 risk of bias assessment results for all studies.

| **Study** | **Risk of Bias** | | | | **Concerns regarding applicability** | | | | **Rationale for Judgement** | |  |
| --- | --- | --- | --- | --- | --- | --- | --- | --- | --- | --- | --- |
| Author; Year | Participants | Index Test | Target Condition | Analysis | | Participants | Index Test | Target Condition | |  | |
| Campbell Arnold, et al. (2022) | ● | ● | ● | ● | | ● | ● | ● | | Overall risk of bias: Low to moderate, prospective study with same day paired imaging comparison to 3T MRI (reference standard), small sample size (*n*=36), recruitment uncertain, only 1 pathology reviewed (MS) limits generalisability to wider populations, uncertainty in independent reporting (bias), images assessed by only n=2 observers | |
| Bauer, et al (2024) | ● | ● | ● | ● | | ● | ● | ● | | Overall risk of bias: Low to moderate, prospective single centre study, paired imaging comparison to 3T MRI (reference standard), small sample size (n=23), recruitment consecutive, limited pathologies reviewed, limits generalisability to wider populations, images assessed by only n=2 observers | |
| Beekman, et al. (2022) | ● | ● | ● | ● | | ● | ● | ● | | Overall risk of bias: Low to moderate, retrospective single centre study, paired imaging comparison to MRI (reference standard), small sample size (n=19), recruitment convenience, limited pathologies reviewed (HIBI), limits generalisability to wider populations, images assessed by only n=1 observer | |
| Cawley et al. (2023) | ● | ● | ● | ● | | ● | ● | ● | | Overall risk of bias: moderate to high, prospective study multi-centre, paired imaging comparison to 3T MRI (reference standard), small sample size (n=87 patients / n=102 scans), recruitment convenience, limited pathologies reviewed (HIBI, strokes, malformations), limits generalisability to wider populations, unblinded images assessed by only n=1 observer (bias) | |
| Cho et al. (2024) | ● | ● | ● | ● | | ● | ● | ● | | Overall risk of bias: Low to moderate, prospective study multi-centre, paired imaging comparison to MRI/CT (reference standard sequences not reported), small sample size (n=50), recruitment convenience, limited pathologies reviewed (HIBI, strokes, ABI), limits generalisability to wider populations, assessed by only n=2 observers, unclear on blinding (bias) | |
| Ciaramitaro et al. (2025) | ● | ● | ● | ● | | ● | ● | ● | | Overall risk of bias: high (due to limited methodology reported), prospective study single centre, paired imaging comparison to 1.5 T MRI (reference standard), very small sample size (n=8), recruitment convenience, limited pathologies reviewed (strokes), limits generalisability to wider populations, unclear reporting (bias) | |
| de Havenon et al. (2023) | ● | ● | ● | ● | | ● | ● | ● | | Overall risk of bias: low to moderate, prospective and retrospective study, paired imaging comparison to 1.5 T MRI (reference standard), sample size (n=124), recruitment convenience, limited pathologies reviewed (WMH), limits generalisability to wider populations, assessed by only n=3 observers | |
| Hilal et al. (2025) | ● | ● | ● | ● | | ● | ● | ● | | Overall risk of bias: Low, prospective study single-centre, paired imaging comparison to MRI/CT (reference standard sequences not reported), small sample size (n=73), recruitment convenience, limited pathologies reviewed (mass lesions, ABI), limits generalisability to wider populations, assessed by only n=2 observers | |
| Kuoy et al. (2022) | ● | ● | ● | ● | | ● | ● | ● | | Overall risk of bias: high, retrospective study single-centre, paired imaging comparison to MRI/CT (reference standard sequences not reported), small sample size (n=36), recruitment convenience, limited pathologies reviewed (stroke, mass lesions, ABI), limits generalisability to wider populations, assessed by only n=1 observer (bias), unclear on blinding during reporting | |
| Lim et al. (2024) | ● | ● | ● | ● | | ● | ● | ● | | Overall risk of bias: low to moderate, retrospective study single-centre, paired imaging comparison to 3T MRI (reference standard), small sample size (n=20), recruitment convenience, limited pathologies reviewed (MS), limits generalisability to wider populations, assessed by only n=2 observers, unclear on blinding during reporting | |
| Mazurek et al. (2021) | ● | ● | ● | ● | | ● | ● | ● | | Overall risk of bias: low to moderate, prospective study single-centre, paired imaging comparison to MRI/CT (reference standard sequences not reported), sample size (n=144), recruitment convenience, limited pathologies reviewed (stroke, ABI), limits generalisability to wider populations, assessed by only n=2 observers | |
| Mazurek et al. (2023) | ● | ● | ● | ● | | ● | ● | ● | | Overall risk of bias: moderate to high, prospective study single-centre, paired imaging comparison to MRI/CT (reference standard sequences not reported), sample size (n=189), recruitment convenience, limited pathologies reviewed (stroke, ABI), limits generalisability to wider populations, assessed by only n=3 observers, unclear on blinding to reporting | |
| Okar et al. (2025) | ● | ● | ● | ● | | ● | ● | ● | | Overall risk of bias: low, retrospective and prospective study single-centre, paired imaging comparison to 3T MRI (reference standard), small sample size (n=55), recruitment convenience, limited pathologies reviewed (MS), limits generalisability to wider populations, assessed by only n=2 observers, explicit blinding during reporting | |
| Okar et al. (2025) | ● | ● | ● | ● | | ● | ● | ● | | Overall risk of bias: high, prospective study single-centre, paired imaging comparison to 3T / 7T MRI (reference standard), very small sample size (n=11), recruitment convenience, limited pathologies reviewed (leukoencephalopathy), limits generalisability to wider populations, assessed by only n=2 observers, explicit blinding during reporting | |
| Sheth et al. (2022) | ● | ● | ● | ● | | ● | ● | ● | | Overall risk of bias: moderate, prospective study single-centre, paired imaging comparison to MRI/CT (reference standard sequences not reported), sample size (n=102), recruitment convenience, limited pathologies reviewed (stroke, ABI), limits generalisability to wider populations, assessed by only n=3 observers, unclear on blinding in reporting | |
| von Danwitz et al. (2025) | ● | ● | ● | ● | | ● | ● | ● | | Overall risk of bias: low to moderate, prospective study single-centre, paired imaging comparison to 1.5T / 3T MRI/CT (reference standard sequences not reported), low sample size (n=17), recruitment convenience, limited pathologies reviewed (stroke), limits generalisability to wider populations, assessed by only n=2 observers, explicit blinding in reporting | |
| Yuen et al. (2022) | ● | ● | ● | ● | | ● | ● | ● | | Overall risk of bias: moderate, prospective study single-centre, paired imaging comparison to 1.5T / 3T MRI/CT (reference standard sequences not reported), low sample size (n=50), recruitment convenience, limited pathologies reviewed (stroke), limits generalisability to wider populations, assessed by only n=2 observers, explicit blinding in reporting, unclear on blinding in reporting | |
